# Combination Therapy as a Promising Way to Fight Oral Cancer

**DOI:** 10.3390/pharmaceutics15061653

**Published:** 2023-06-04

**Authors:** João P. N. Silva, Bárbara Pinto, Luís Monteiro, Patrícia M. A. Silva, Hassan Bousbaa

**Affiliations:** 1 UNIPRO—Oral Pathology and Rehabilitation Research Unit, University Institute of Health Sciences (IUCS), Cooperativa de Ensino Superior Politécnico e Universitário (CESPU), Rua Central de Gandra, 1317, 4585-116 Gandra, Portugal; joaosilva_06@hotmail.com (J.P.N.S.); barbara_fernades_pinto@hotmail.com (B.P.); luis.monteiro@iucs.cespu.pt (L.M.); 2 TOXRUN—Toxicology Research Unit, University Institute of Health Sciences (IUCS), Cooperativa de Ensino Superior Politécnico e Universitário (CESPU), Rua Central de Gandra, 1317, 4585-116 Gandra, Portugal

**Keywords:** oral cancer, signaling pathways, targeted therapy, anticancer drugs, combination therapy

## Abstract

Oral cancer is a highly aggressive tumor with invasive properties that can lead to metastasis and high mortality rates. Conventional treatment strategies, such as surgery, chemotherapy, and radiation therapy, alone or in combination, are associated with significant side effects. Currently, combination therapy has become the standard practice for the treatment of locally advanced oral cancer, emerging as an effective approach in improving outcomes. In this review, we present an in-depth analysis of the current advancements in combination therapies for oral cancer. The review explores the current therapeutic options and highlights the limitations of monotherapy approaches. It then focuses on combinatorial approaches that target microtubules, as well as various signaling pathway components implicated in oral cancer progression, namely, DNA repair players, the epidermal growth factor receptor, cyclin-dependent kinases, epigenetic readers, and immune checkpoint proteins. The review discusses the rationale behind combining different agents and examines the preclinical and clinical evidence supporting the effectiveness of these combinations, emphasizing their ability to enhance treatment response and overcome drug resistance. Challenges and limitations associated with combination therapy are discussed, including potential toxicity and the need for personalized treatment approaches. A future perspective is also provided to highlight the existing challenges and possible resolutions toward the clinical translation of current oral cancer therapies.

## 1. Introduction

Head and neck cancer is the seventh most prevalent cancer worldwide, and it includes tumors of the oral cavity, salivary glands, nasopharynx, and oropharynx [1,2]. According to GLOBOCAN, around 380,000 new lip and oral cavity cancer (OCC) cases and 180,000 deaths were reported in 2020, making it the most common form of head and neck cancer [1]. Oral squamous cell carcinomas (OSCCs) comprise nearly 90% of all oral cancers. OSCCs have an epithelial origin, but the mechanism that causes the transition from normal cells to cancer cells is still unclear [3,4]. The main factors associated with the emergence of OSCC are tobacco and alcohol consumption, among other factors, such as genetic and epigenetic, as well as human papillomavirus (HPV), a recognized etiologic factor mainly associated with oropharyngeal cancers, and Epstein–Barr virus infections [3,5]. While OSCCs are more prevalent in developing countries, there has been a reported increase in developed countries, as well. This rise has been attributed to changes in lifestyle activities [6].

In recent years, there has been a small improvement in the survival of patients with oral cancer, largely due to the use of radiation or chemoradiation therapy after surgery, combined with advances in clinical procedures [7,8]. However, delays in initiating treatment prevent greater improvement [9]. The treatment of oral cancer is dependent on type, location, and cancer stage, with early diagnosis leading to a better prognosis [2,10]. Early detection of cancer significantly increases the chance of successful surgery or radiotherapy treatment [11]. However, treatment failure is often attributed to local recurrence, lymph node metastasis, and drug resistance [12,13]. Consequently, there is a need for new therapeutic approaches and/or the development of new drugs. Nevertheless, developing new drugs for cancer treatment is a costly and time-consuming process that requires pre-clinical in vitro and in vivo assays, followed by clinical trials. On average, this translates into more than 12 years of research before new drugs can be commercialized [14]. 

Although still quite common in cancer treatment, chemo-monotherapy modalities are considered less effective than combination therapies. Monotherapies usually have non-selective targets that lead to death of both tumor and healthy cells [9,15]. Furthermore, cancer cells can develop resistance to the treatment. Regardless of cross-resistance, therapeutic combinations can lead to a prolonged response [16]. Although combinatorial modalities also present toxicity, the targeting of different pathways helps attenuate this issue. In addition, therapeutic combinations can also lead to synergistic or additive effects, which allow for a reduction in drug dosages [15,17]. Therefore, therapeutic combinations and drug repurposing present themselves as alternatives that have been intensively explored in recent years. Although some promising pre-clinical results have been reported, there is still a minor impact on clinical treatment, but hopefully, this will change in the near future. Here, we propose to review the therapeutic combinations currently in use in the treatment of oral cancer, the therapeutic combinations with promising results in preclinical trials, and the therapeutic combinations that reached clinical trials. The review is focused on the important pathways commonly targeted in oral cancer therapy.

## 2. Current Therapeutic Options for Oral Cancer Treatment

The therapeutic options for oral cancer are largely based on the tumor stage (extent) according to Tumor, Lymph node, and Metastasis classification from the American Joint Committee on Cancer [18,19]. Nonetheless, other factors also need to be considered. For instance, HPV-related tumors have a better prognosis than non-related ones and are more responsive to chemoradiotherapy and immune checkpoint inhibition [20]. 

Conventional strategies include surgery, radiation therapy, and chemotherapy, either alone or in combination (Table 1). However, surgery remains the standard treatment modality for oral cancer, either alone or in combination with other therapy approaches. The FDA-approved drugs mostly used in chemotherapy that can be given with or without gamma ray radiation include Cisplatin, Carboplatin, 5-fluorouracil (5-FU), Hydroxyurea, Paclitaxel, and Docetaxel [21]. The first four are DNA-targeting agents, while Paclitaxel and Docetaxel target microtubules [22]. Methotrexate, Bleomycin, and Capecitabine are less commonly used and belong to the DNA-targeting family. In addition to traditional options, immunotherapy has gained more attention, and drugs include monoclonal antibodies (mAbs), such as Cetuximab and Nimotuzumab, and immune checkpoint inhibitors, such as Pembrolizumab and Nivolumab. Cetuximab binds to the epidermal growth factor receptor (EGFR), while Pembrolizumab and Nivolumab are anti-programmed cell death protein 1 (PD-1) inhibitors [23]. 

Patients with cancer in more advanced stages, namely, recurrent or metastatic head and neck squamous cell carcinoma (R/M HNSCC) or locally advanced head and neck cancer (LA-HNSCC), are usually treated with combinatorial therapies, including surgery, radiotherapy, and chemotherapy, mainly with the purpose of prolonging remission [2]. A combination of chemoradiotherapy and Cisplatin, the first FDA-approved platinum drug for cancer treatment, is the standard treatment for LA-HNSCC patients. However, Cetuximab in combination with radiotherapy is used when patients are considered unfit for the standard treatment [24]. A recent study comparing various modalities of treatment for LA-HNSCC reported that hyperfractionated radiotherapy with concomitant chemotherapy seemed to be the best treatment option [25]. Regarding combinatorial chemotherapy, the so-called “EXTREME” regimen, a combination of Cetuximab with platinum and 5-FU, followed by maintenance of Cetuximab, is used as first-line treatment for R/M HNSCC patients since it was shown to improve overall survival (OS) [5,24]. For patients that cannot be subjected to the EXTREME regimen, treatment with a taxane, such as Paclitaxel, or Cisplatin plus Cetuximab, or the combination of Gemcitabine and Paclitaxel, can be used as first-line treatment [21,26]. Induction chemotherapy (IC) efficacy in oral cancer needs further assessment, but it can be an option for selected patients [21]. Nonetheless, for LA-HNSCC, IC with Docetaxel-5-FU-Cisplatin has been approved [27].

Pembrolizumab or Nivolumab are anti-PD-1 mAbs recently approved in the United States and can be used individually or combined with a platinum and 5-FU for R/M HNSCC patients. They will probably become the first line of treatment in the next few years [2,28].

Despite various therapeutic options being available, only small improvements in the survival rate of patients with advanced disease have been reported, and, thus, new therapies and/or improvements of the current therapies are needed. 

**Table 1:** Main advantages and disadvantages of the existent treatment modalities in oral cancer.

Therapies	Advantages	Disadvantages	References
Surgery	- Gold standard for managing oral cancers, especially for early-stage tumors; - Possibility of complete tumor with clear margins removal, minimizing the chance of recurrence; - Suitable for debilitated patients or those with advanced age.	- Can be dramatic, painful, and have wound-healing issues and surgical complications, such as infection and increasing recovery time; - Resection may affect head and neck function, leading to breathing, speaking, or swallowing difficulties; - Depending on tumor staging and location, aesthetic reconstruction surgery may be necessary; - Cost of 2-year follow-up (only treatment): USD 8320 ± 15,111.	[29,30]
Radiotherapy	- Suitable for treatment of inoperable tumors or tumors that have spread; - Non-invasive treatment option; - Can be used as an alternative to surgery or combined with surgery to minimize the amount of surgery required.	- Radiation-induced side effects can be uncomfortable, painful, and long-lasting, including trismus, nerve injury, xerostomia, hypothroidism, osteorarionecrosis of maxilla and mandible, oral mucositis; - Patients are typically required to attend multiple appointments for several weeks. - Cost of 2-year follow-up (only treatment): USD 50,362 ± 28,928.	[30,31,32]
Chemotherapy	- Suitable to treat tumors that have spread and cannot be treated with surgery or radiation therapy alone; - Cost of 2-year follow-up (only treatment): USD 3277 ± 2822.	- Associated with a wide range of colateral effects (nausea, vomiting, immunosuppression, fatigue), affecting patients’ quality of life; - It requires hospitalization for administration and medical management of side effects.	[30,33]

## 3. New Combinatorial Approaches for Oral Cancer Treatment

Despite stepwise advances in our understanding of the molecular basis of oral carcinogenesis, the complexity and diversity of pathways and mechanisms that drive cancer progression and invasion may compromise the success of the treatment when used in a monotherapy approach. Therefore, combinatorial modalities that simultaneously target different pathways are expected to result in more clinical benefit for patients. Consequently, several combinatorial strategies have been explored in preclinical and clinical trials, and their results will be summarized below. Our analysis will specifically focus on combinations involving key pathways commonly targeted in cancer therapy and explored in combinatorial approaches, namely, DNA damage, the epidermal growth factor receptor, cyclin-dependent kinases, epigenetic readers, immune checkpoint proteins, and microtubules.

### 3.1. DNA Damage Response Inhibition-Based Combination Therapies

**Figure 1:**
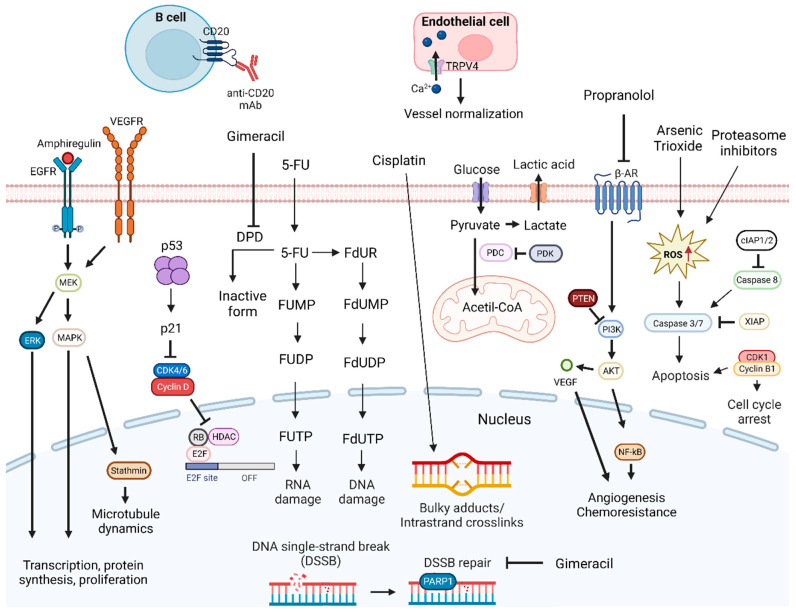
DNA-damage-targeting drug mechanisms and possible synergistic co-targeted pathways. DNA-damaging drugs induce cell death signaling. Drugs causing DNA damage combined with TRPV4 agonist, inhibitors of DNA repair mechanisms, inducers of cell death signaling, anti-CD20 mAbs, or inhibitors of PDK, PI3K, MAPK, ERK, EGFR, VEGFR, and CDKIs proteins can enhance antitumoral effects. Abbreviations: 5-FU, 5-fluorouracil; β-AR, β-adrenergic receptor; CDK, cyclin-dependent kinase; CDKIs, CDK inhibitors; cIAP1/2, cellular inhibitor of apoptosis protein 1/2; DPD, dihydropyrimidine dehydrogenase; EGFR, epidermal growth factor receptor; ERK, extracellular signal-regulated kinases; FdUDP, fluorodeoxyuridine diphosphate; FdUMP, fluorodeoxyuridine monophosphate; FdUR, fluorodeoxyuridine; FdUTP, fluorodeoxyuridine triphosphate; FUDP, fluorouridine diphosphate; FUMP, fluorouridine monophosphate; FUTP, fluorouridine triphosphate; HDAC, histone deacetylase; MAPK, mitogen-activated protein kinases; MEK, MAPK/ERK kinase; NF-κB, nuclear factor κB; PARP1, poly (ADP-ribose) polymerase 1; PDC, pyruvate dehydrogenase complex; PDK, pyruvate dehydrogenase kinase; PTEN, phosphatase and tensin homolog; Rb, retinoblastoma protein; RNA, ribonucleic acid; ROS, reactive oxygen species; TRPV4, transient receptor potential vanilloid 4; VEGF, vascular endothelial growth factor; VEGFR, vascular endothelial growth factor receptor; XIAP, x-linked inhibitor of apoptosis protein. Created by the authors with BioRender.com.

As DNA molecules can experience harmful lesions, cells have developed DNA damage response (DDR) pathways to address such damage. While DDR pathways promote genomic stability in normal cells, they can also protect cancer cells from DNA lesions, particularly those caused by external DNA-damaging agents [34]. Currently, there are several therapeutic options for cancer treatment that aim for the disruption of DNA damage repair mechanisms, inhibition of DNA replication, and damaging of cancer cells DNA to induce cell death signaling (Figure 1) [35]. However, DNA targeting drugs usually have severe side effects since they lack tumor specificity. Additionally, cancer cells develop resistance to therapy, and, therefore, there is a need to find ways to overcome these issues [36]. Moreover, there is a need to improve the effectiveness of DNA-targeting drugs, namely, through combination therapies (Table 2). 

For this purpose, a system comprised of polylactic acid (PLA) nanoparticles loaded with Cisplatin–chloroquine was investigated [37]. Cisplatin is a platinum-based anticancer drug that inhibits the proliferation of cancer cells by binding to guanine and adenine, forming adducts and triggering DNA damage [38]. Consequently, DNA replication is inhibited, and the cell cycle is arrested [39]. As a consequence, activation of DNA repair mechanisms occurs, but due to the inflicted damage, cell death signaling is triggered. Chloroquine inhibits autophagy, a pro-tumoral process shown to be associated with Cisplatin resistance. This PLA combination led to OSCC proliferation inhibition through apoptosis and oxidative stress [39,40]. A system with transfersomes loaded with both 5-FU and a cyclooxygenase-2 (COX-2) inhibitor was also investigated. 5-FU is an antimetabolite fluoro-deoxynucleotide analogous to uracil that inhibits thymidylate synthase (TS), leading to apoptosis, and is commonly used in oral cancer treatment [41]. It is converted into fluorodeoxyuridine monophosphate (FdUMP), fluorodeoxyuridine triphosphate (FdUTP), and fluorouridine triphosphate (FUTP) after cellular uptake. FdUMP forms an irreversible ternary complex with TS and the folate cofactor 5,10-Methylenetetrahydrofolate, inhibiting the conversion of deoxyuridine monophosphate to deoxythymidine monophosphate. FdUTP is incorporated in the DNA causing direct damage, whereas FUTP is directly incorporated into RNA preventing pre-ribosomal RNA maturation [42,43]. COX-2 is associated with carcinogenesis and cancer cells resistance to therapy [44]. The combination showed synergistic effects, while increasing drug delivery to cancer cells [44]. 

In breast cancer, the combination of Cisplatin with a Poly (ADP-ribose) polymerase (PARP) inhibitor led to enhanced cytotoxicity [45]. PARP-1 is involved in base excision repair, DNA replication, and genomic maintenance, and its inhibition causes the accumulation of DNA double-strand breaks (DSB) [46,47]. In a similar combination in OSCCs, a synergistic effect in vitro and enhanced suppression of in vivo tumor growth were described [47]. In addition, it is suggested that treatment with a PARP inhibitor could prevent drug resistance. In a phase 1 trial, the addition of a PARP inhibitor to Cisplatin and Paclitaxel IC was well tolerated and led to low toxicity [48]. Therapeutic combinations with natural products in the treatment of several types of cancer have shown promising anticancer activity [49]. For instance, the combination of PARP inhibitors with curcumin, a plant-derived bioactive molecule that interferes with DNA damage repair mechanisms and shows anticancer activity, was explored. The results demonstrated that indirect NECTIN 4, a member of the cellular adhesion molecule family, deregulation enhanced anti-angiogenic activity, and increased cell death was achieved mainly by trapping PARP-1 at DNA damage sites [50,51]. Recently, a combination of two PARP inhibitors enhanced apoptotic effects in curcumin pre-treated oral cancer cells by deregulating the base excision repair pathway [52].

Arsenic trioxide (ATO) was shown to exert anticancer activity in different types of tumors, although high concentrations are needed to achieve this outcome [53,54,55]. In OSCC cells, the combination of ATO with Cisplatin led to synergistic anticancer effects, probably through apoptosis induction due to caspase-3/7 signaling pathway activation [56]. Furthermore, this combination showed a synergistic effect in head and neck cancer initiating cells allowing a drug dose reduction [57]. A nitrated [6,6,6]tricycle derivative, SK2, with a dioxabicyclo[3.3.1]nonane core that can be found in various natural products has been shown to exert antiproliferative activity [58]. In oral cancer cells, a combination with Cisplatin enhanced apoptosis, possibly through induction of oxidative stress [59].

The ubiquitin–proteasome pathway is essential for protein degradation, and its inhibition increases ROS production, leading to DNA damage and promotion of apoptosis [60]. In OSCC, different proteasome inhibitors in combination with Cisplatin enhanced apoptosis through dissociation of the E-cadherin/β-catenin complex essential for cell adhesion [60]. Recently, proteasome inhibitors were shown to sensitize OSCC to Cisplatin through the ROS/DNA damage/p53 axis [61]. Gimeracil is known to enhance the antitumor effects of 5-FU and improve radiotherapy efficacy by inhibiting DNA repair pathways. It also promotes the anticancer activity of Camptothecin and Hydroxyurea and enhances heat sensitivity in OSCCs [62,63]. The combination of Gimeracil with Cisplatin suppressed OSCC tumor growth both in vitro and in vivo, possibly by more effectively inhibiting the DNA DSB repair system than Cisplatin alone [64].

Despite 5-FU being commonly used, HNSCC cells can develop resistance. As a result, therapeutic approaches capable of resensitizing cancer cells to 5-FU have been explored. For instance, an extract from Juniperus communis, a plant used in traditional Chinese medicine, demonstrated a synergistic effect by enhancing proliferative inhibition when used in combination with 5-FU. In the oral cancer cell line OECM-1, this combination led to cell cycle arrest, probably due to p53-p21-retinoblastoma protein (Rb) signaling modulation [65]. Azurin, a protein produced by Pseudomonas aeruginosa that can induce apoptosis or lead to cell cycle arrest, has also been shown to increase OSCC sensibility to 5-FU and Etoposide, a topoisomerase II inhibitor [66]. In these combinatorial therapies, the increased sensitivity was dependent on increased cyclin B1 expression induced by azurin, even though the molecular mechanism is still poorly understood. Coincidentally, decreased levels of cyclin B1, Cdc25C, and Cdk1 induced by a fatty acid synthase inhibitor sensitized SCC-9 LN-1 cells to both Cisplatin and Paclitaxel, a microtubule inhibitor, but not to 5-FU [67]. Propofol, an intravenous anesthetic agent, exhibited anticancer activity in different types of cancer [68,69]. In oral cancer, it can overcome 5-FU resistance by downregulating amphiregulin, a growth factor associated with a poor prognosis, which seems to reduce EGFR activation [70].

Propranolol is used to treated several conditions, such as hypertension, and it was shown to promote antitumor activity in various types of cancer [71,72]. It acts by binding to β-adrenergic receptors, inhibiting their activation. In OSCC, it was shown to enhance the antitumor effects of 5-FU and Cisplatin. It is suggested that the enhancement of 5-FU anticancer activity is due to increased phosphatase and tensin homolog (PTEN) expression. PTEN regulates PI3K activation, which is involved in angiogenesis and cell survival [73].

The Docetaxel, Cisplatin, and Fluorouracil (TPF) regimen is a treatment option reported to reduce tumor volume and facilitate radical surgery [74]. However, overexpression of stathmin, a crucial regulator of the cell cytoskeleton, promotes proliferation and decreases cancer cells’ responsiveness to the TPF regimen, making stathmin expression a predictor of the outcome of this treatment in OSCC patients. Additionally, the PI3K/AKT/mTOR pathway is involved in the regulation of stathmin expression and phosphorylation and was reported to be active in OSCC patients. Consequently, inhibition of PI3K leads to resensitization of OSCC cells to the TPF treatment by reducing stathmin expression and phosphorylation and enhancing apoptosis, while slowing tumor growth [75]. In clinical trials, the combination of Cisplatin with inhibitors of the PI3K/AKT/mTOR pathway was deemed feasible, with one study showing promising OS and progression-free survival (PFS) rates [76,77].

Vascular endothelial growth factor (VEGF) expression in OSCC is associated with a poor prognosis since it plays a crucial role in angiogenesis, which is critical for tumor growth and metastization [78,79]. In other types of cancer, a combination of S-1 with Bevacizumab showed modest to high efficacy [80,81]. S-1 is a fluoropyrimidine combination of three compounds: Tegafur, Gimeracil, and Oteracil potassium. Tegafur is an anticancer prodrug that gets metabolized to 5-FU and interferes with RNA and represses DNA synthesis. Gimeracil is a dihydropyrimidine dehydrogenase inhibitor that prevents 5-FU from being metabolized, and Oteracil inhibits 5-FU phosphorylation in the gastrointestinal mucosa to reduce toxicity [82]. Bevacizumab is an anti-VEGF antibody with antiangiogenic activity in the tumoral microenvironment, while promoting vascular normalization, allowing for better delivery of drugs into tumors [83]. A synergistic effect in OSCCs was noticed for the combination of S-1 with Bevacizumab, leading to cell proliferation inhibition. In addition, enhancement of apoptosis was observed in vivo. Nevertheless, a combination of 5-FU and Bevacizumab only inhibited cell proliferation, with no synergy [83]. In a phase 2 trial, the addition of Bevacizumab to Cisplatin and Gemcitabine, a nucleoside analog that interferes with DNA synthesis, in patients with nasopharyngeal carcinoma (NPC) was well tolerated, and promising anticancer activity was observed [84]. Transient receptor potential vanilloid 4 (TRPV4) channels in endothelial cells have also been associated with angiogenesis. These channels are usually repressed in tumor endothelial cells [85]. Recently, in an animal model of OSCC, a combination of a TRPV4 agonist with Cisplatin led to vessel normalization, possibly via increased expression of both angiopoietin-1 (Ang-1), an angiogenic factor, and Tie-2, a tyrosine kinase receptor activated by Ang-1 [86,87].

The tumor immune microenvironment can promote resistance to treatment. For instance, in a SCC mouse model, depletion of CD20+ B cells enhanced the tumor response to platinum and taxane treatment [88]. A phase I trial with R/M HNSCC patients explored a similar treatment option with the combination of an anti-CD20 mAb with Cisplatin and Gemcitabine, which was deemed safe, although no clear benefit could be ascertained [89].

**Table 2:** Main combination therapies based on DNA damage targeting used in clinical and preclinical trials for oral cancer treatment.

Combination Therapies	Study Design/Treatment Type	Cancer Type and Stage	Main Reported Outcome	References
PLA nanoparticles loaded with Cisplatin–chloroquine	Preclinical trials	N/A	Inhibited OSCC proliferation through oxidative stress and apoptosis.	[39,40]
Transfersomes loaded with 5-FU and COX-2 inhibitors	Preclinical trial	N/A	Exerted synergistic effects, increasing drug delivery efficiency.	[44]
Cisplatin plus Gemcitabine and Rituximab	Phase 1 clinical trial Patients previously treated	- R/M HNSCC	The treatment was considered safe, but no clinical benefit could be ascertained.	[89]
Cisplatin with a PARP inhibitor	Preclinical trial	N/A	Exerted synergistic effects in vitro and potentiated in vivo tumor growth suppression.	[47]
Combination of a PARP inhibitor with Cisplatin and Paclitaxel	Phase 1 clinical trial 1st line (IC)	LA-HNSCC IVA-B	Displayed low toxicity and was well tolerated.	[48]
PARP inhibitors combined with Curcumin	Preclinical trials	N/A	Enhanced anti-angiogenic activity and increased cell death.	[50,51]
ATO combined with Cisplatin	Preclinical trial	N/A	Exerted synergistic effects, increasing OSCC apoptosis.	[56]
Gimeracil combined with Cisplatin	Preclinical trial	N/A	Inhibited in vitro and in vivo OSCC tumor growth	[64]
Combination of 5-FU with Juniperus communis extract	Preclinical trial	N/A	Exerted synergistic effects, potentiating proliferative inhibition.	[65]
Azurin combined with 5-FU or Etoposide	Preclinical trial	N/A	Increased OSCC sensibility, improving anticancer response.	[66]
Propofol combined with 5-FU	Preclinical trial	N/A	Reduced pharmacological resistance of oral cancer cells.	[70]
Combination of Cisplatin with PI3K/AKT/mTOR pathway inhibitors and radiotherapy	Phase 1 clinical trials -	LA-HNSCC ≥III	Exhibited satisfactory OS and PFS rates.	[76,77]
Combination of S-1 with Bevacizumab	Preclinical trial	N/A	Exerted synergistic effects, causing in vitro cell proliferation inhibition, and exacerbated apoptosis in vivo.	[83]
Combination of 5-FU with Bevacizumab	Preclinical trial	N/A	Exerted no synergistic effects, but was able to reduce cell proliferation.	[83]
Bevacizumab combined with Cisplatin and Gemcitabine	Phase 2 clinical trial -	LA NPC III–IVc	Displayed anticancer activity and was well tolerated.	[84]
Xevinapant, Cisplatin, and radiotherapy	Phase 1/2 clinical trials -	LA-HNSCC III/IVa/IVb	Showed improved efficacy. A 5-year follow-up demonstrated improved 5-year OS and 3-year PFS, and was deemed safe.	[90,91]
Dichloroacetate, Cisplatin, and radiotherapy	Phase 2 clinical trial 1st line	LA-HNSCC III/IVa/IVb	The treatment was safe, but the efficacy could not be determined.	[92]
Gemcitabine in combination with Nedaplatin and radiotherapy	Clinical trial -	R/M or LA-HNSCC III–IV	Can be a therapeutic option for HNSCC, although high number of adverse events highlights the need for the optimization of dose and schedule.	[93]
Combination of Bortezomib, Camptothecin, and Doxorubicin	Preclinical trial	N/A	Potentiated cytotoxicity only in KB oral cancer cells and not in non-cancerous cells.	[94]
Postoperative weekly administration of Cisplatin plus radiation	Phase 2/3 clinical trial Postoperative	LA-HNSCC III/IVa/IVb	Similar efficacy, but less toxicity when compared with 3-weekly Cisplatin administration.	[95]
One cycle chemoselection split-dose TPF IC before two cycles of split TPF followed by curative surgery combined with postoperative radiotherapy	Phase 2 clinical trial -	LA OPSCC and LA OCC III/IVa	Well tolerated and a good strategy to select patients that will benefit with TPF treatment.	[96]
TPF as induction chemotherapy	Phase III clinical trial 1st line (IC)	LA OSCC -	Did not improve survival of unselected patients, but patients which achieved FPR had good OS and PFS.	[97]

Abbreviations: ATO, arsenic trioxide; COX-2, cyclooxygenase-2; FPR, favorable pathological response; IC; induction chemotherapy; LA OCC, locally advanced oral cavity cancer; LA OPSCC, locally advanced oropharyngeal squamous cell carcinoma; LA OSCC, locally advanced oral squamous cell carcinoma; N/A, not applicable; OS, overall survival; OSCC, oral squamous cell carcinoma; PARP, poly (ADP-ribose) polymerase; PFS, progression-free survival; PLA, polylactic acid; -, data not provided.

Strategies to improve the combination of radiotherapy and platinum have been explored. For instance, addition of Xevinapant, an inhibitor of the antiapoptotic proteins XIAP and cIAP1/2, to this combination led to improved efficacy [90]. A follow-up showed improved 5-year OS and 3-year PFS, and was deemed safe [91].

Pyruvate dehydrogenase kinase (PDK) is involved in the promotion of an acidic and immunosuppressive tumor microenvironment by promoting lactate production [92]. In a phase II study, addition of a PDK inhibitor to Cisplatin plus radiotherapy for LA-HNSCC was deemed safe, although the efficacy could not be ascertained [92].

A combination of Gemcitabine, Nedaplatin, and radiotherapy for recurrent or LA-HNSCC showed an ORR of 100%, a 1-year OS of 75%, and a PFS of 66.7%. However, the treatment option led to a high rate of adverse events [93].

In addition to new therapeutic combinations, a focus on improving those currently in use is necessary. Occasionally, drug doses and/or ratios must be optimized to achieve better results. By reducing the drug concentration, less toxicity is expected, with the possibility of synergistic effects that could enhance anticancer activity. For instance, the combination of Bortezomib, Camptothecin, and Doxorubicin, which were being used close to the maximum tolerated doses, revealed that the optimized therapy had enhanced cytotoxicity in KB oral cancer cells, but not in control cells [94]. In a phase 2/3 clinical trial, postoperative weekly administration of Cisplatin plus radiation was compared with 3-weekly Cisplatin chemoradiotherapy for LA-HNSCC patients. Both approaches had a similar efficacy, but with a better safety profile for the weekly administration of Cisplatin [95].

Usually, radiotherapy or radiochemotherapy are used after surgery, but approaches with IC followed by surgery have also been explored. A two-arm multicenter phase 2 trial, intended to assess if a split-dose of IC with TPF before surgery was effective for locally advanced (LA) resectable oral and oropharyngeal cancer, showed that this strategy permits fast selection of patients that can benefit from TPF, while sparing toxicity for patients that do not respond to the therapy [96]. The PFS rate, which comprises the period from the beginning of treatment to the reappearance of the disease or even death for patients that responded to the therapy, was 88.5%, while the non-responders showed a smaller rate of 60.6%. However, in a phase 3 trial, the survival of patients with LA-OSCC did not improve. Yet, patients that achieved a favorable pathological response (FPR) following treatment had considerable improvement in survivability when compared to both the control group and patients that did not achieve FPR [97].

Neoadjuvant TPF was assessed for patients with OCC and showed a lower ORR than previously observed in other studies. This may be due to the fact that other studies were more heterogeneous and had a low number of patients with OCC. A link between the nodal stage and therapeutic response was also found, where bulky nodes or lymph nodes with necrosis may lead to lower response to treatment. Nonetheless, fewer progression events and better survival was observed in patients with complete or partial responses [98].

Overall, combinations with DNA-targeting drugs seem to have promising results, proving that it is possible to improve specific targeting of cancer cells, increasing the anticancer activity of several drugs, and overcome resistance to therapy. Furthermore, the results from clinical trials seem to corroborate these findings. These combinations should continue to be explored even more since some of these drugs are among the most commonly used in the treatment of oral cancer.

### 3.2. Epidermal Growth Factor Receptor Inhibition-Based Combination Therapies

**Figure 2:**
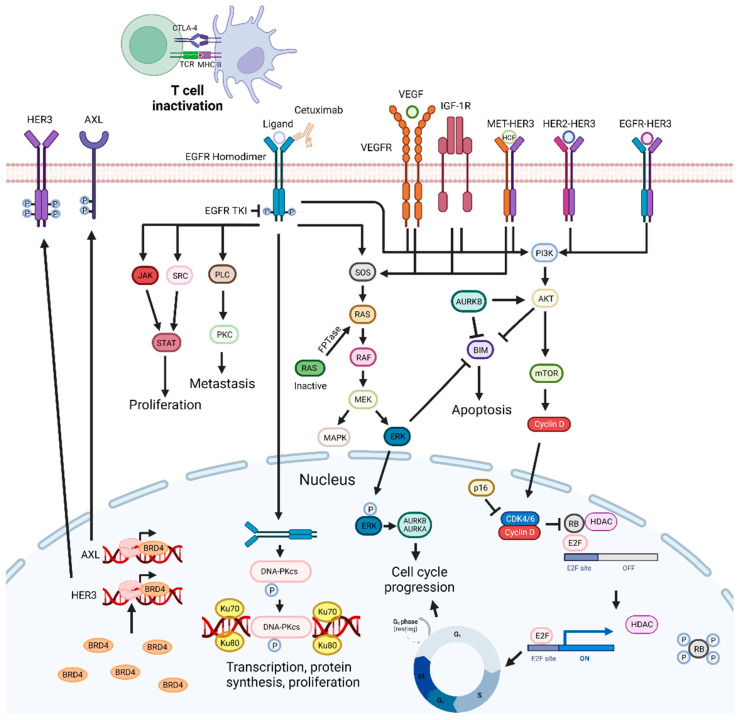
EGFR-targeting drug mechanisms and possible synergistic co-targeted pathways. Inhibition of the EGFR signaling pathways impairs cell proliferation and metastization, and can trigger apoptosis. EGFR inhibitors can be combined with PI3K/AKT, ERK, CDKs, and VEGF inhibitors, radiotherapy, BET inhibitors, Aurora kinase inhibitors, drugs targeting DNA repair pathways, c-Met inhibitors, CTLA-4, SRC, FTPase, NF-κB, IGF1R, and HDAC inhibitors, leading to synergistic effects. Abbreviations: AURKB, aurora kinase B; BRD4 bromodomain-containing protein 4; CDK, cyclin-dependent kinase; CTLA-4, cytotoxic T-lymphocyte-associated protein 4; EGFR, epidermal growth factor receptor; FTPase, farnesyltransferase; IGF-1R, insulin-like growth factor 1 receptor; Rb, retinoblastoma protein; RTKs, receptor tyrosine kinases; TKI, tyrosine kinase inhibitor; VEGF, vascular endothelial growth factor; VEGFR, vascular endothelial growth factor receptor. Created by the authors with BioRender.com.

EGFR, human epidermal growth factor receptor-type 2 (HER-2), and HER-3 belong to the EGFR family. These tyrosine kinase receptors are involved in several pro-tumoral processes, such as tumor growth and metastasis [99,100,101]. They are found to be overexpressed in HNSCC, with EGFR being overexpressed in 42% to 80% of these tumors [99,100]. Overexpression of EGFR is usually associated with a poor prognosis [99]. Binding of ligands to EGFR leads to homodimer or heterodimer formation, subsequently leading to the phosphorylation of the C-terminal tail [102,103]. Since these receptors exhibit differences in the C-terminal domains, a single ligand can lead to the activation of several signaling pathways [102]. EGFR is involved in the activation of the RAS/RAF/MAPK/ERK, JAK-STAT, and SRC pathways, which are associated with proliferation; the PLC-γ1-PKC pathway, involved in metastization; and the PI3K/AKT pathway, directly or through adaptor proteins, which is involved in proliferation and inhibition of apoptosis [99,101,103,104]. EGFR also regulates autophagy and metabolism, which can be advantageous for the survival and development of therapeutic resistance in tumor cells [105]. Thus, therapeutic targeting of EGFR in HNSCC has been widely explored and with some success since, as mentioned above, Cetuximab is one of the few drugs approved for the treatment of this disease (Figure 2) [106,107].

In the context of oral cancer, two approaches for inhibiting EGFR are commonly employed: tyrosine kinase inhibitors (TKIs) and mAbs [108]. These approaches differ in their mechanisms of action and patient selection criteria. TKIs, such as small molecule drugs, directly target the intracellular kinase domain of EGFR, inhibiting the intracellular signaling pathway. Anti-EGFR mAbs bind to the extracellular domain of the receptor, preventing ligand binding and receptor activation. Moreover, mAbs have the ability to activate, complement, and induce ADCC. Compared to mAbs, TKIs exhibit lower target specificity and have a shorter half-life [109,110,111]. In addition, overexpression of HER family proteins could enhance EGFR activation, even in the presence of EGFR-specific TKI. Therefore, inhibiting HER2 could be a promising strategy for better EGFR TKI activity. Cells exhibiting the highest levels of EGFR demonstrated greater sensitivity to TKIs than to mAbs. Thus, when choosing between TKIs and mAbs, these factors should be taken into account, as well as the different patient-specific factors, such as primary tumor dimension, thickness and margin status, the patient’s overall health, and the stage and location of the cancer [112,113]. Nonetheless, EGFR tumor detection can show some limitations. For instance, immunohistochemistry is commonly used for the detection of EGFR, but fails to consistently identify mutations or distinguish between receptors with high or low affinity. Consequently, this limitation may contribute to the observed absence of correlation between EGFR expression levels and the response to EGFR-targeting therapies [114]. mAbs targeting EGFR have been reported to induce NK-dependent ADCC, while activating CD8+ T-cell responses [115,116]. Therefore, it is suggested that low levels of lymphocytes can have an impact on mAbs efficacy. In this sense, a recent study reported that a higher neutrophil/leucocyte ratio was correlated with a lower OS in R/M HNSCC patients treated with cetuximab-based chemoimmunotherapy [117]. In addition, the composition of the tumor immune infiltrate is also predictive of the general treatment response. For instance, PD-1+ and TIM-3+ CD8+ TILs augmented frequencies correlated with a worse clinical outcome in patients treated with Cetuximab [118]. Moreover, higher frequencies of CD8+CD28+ T cells and a lower frequency of CD8+CD28− T cells can lead to lower therapy responses in HNSCC patients [119].

In HNSCC, EGFR inhibition has a supra-additive effect in combination with irradiation with a phase 3 clinical trial, pointing to significant improvements in the OS of patients with LA-HNSCC [120,121]. A recent retrospective study focusing on this combination for OCSCC showed it was slightly less effective than for HNSCC patients, but with a good safety profile and efficacy [122]. Therefore, the possibility of enhancing the effect of this combination with the addition of other drugs has been investigated (Table 3).

A therapeutic strategy explored was the inhibition of the PI3K/AKT signaling pathway associated with tumor progression and resistance to EGFR inhibition and radiotherapy [108,120,123]. For instance, PI3K/AKT is involved in cell survival through DNA-dependent protein kinase (DNA-PK) activity, which is responsible for repair mechanisms, and hypoxia, a mechanism of radiotherapy resistance. In HNSCC, blocking of PI3K/AKT signaling improved oxygenation [124]. Radiosensitization of HNSCC tumor cells using PI3K inhibitors has been widely reported. PF-05212384, a dual PI3K/mTOR inhibitor, and Taselisib and LY294002, two PI3K inhibitors, were all shown to improve tumor cell radiosensitivity [125,126,127]. Cetuximab is a mAb that inhibits EGFR activation, consequently leading to inhibition of AKT phosphorylation. Mutations that activate PI3K can lead to resistance to Cetuximab. Nonetheless, PI3K inhibition can restore cell sensitivity to this drug [108]. However, clinical trials exploring this combination showed no improvement in PFS, objective response rate (ORR), or OS, probably due to the fact that patients lacked the genetic alterations that are considered to be predictive of response to treatment with PI3K inhibitors, or were early terminated due to high toxicity [128,129]. More recently, this approach concurrent with radiation inhibited the activation of the MAPK pathway induced by radiotherapy. In clinical trials, this type of combination showed anticancer activity and was deemed safe and should be explored [120,130,131]. Receptor tyrosine kinase (RTK) overexpression is also associated with Cetuximab resistance [132]. Expression of these receptors is dependent on the bromodomain-containing protein 4 (BRD4), which is involved in the regulation of transcription [133]. Inhibition of BRD4 leads to a decrease in RTK expression [132,134]. In patient-derived tumor xenograft (PDTX), a delay in tumor outgrowth and a reduction of RTK signaling were observed [132].

EGFR targeting causes impairment of DNA DSB repair by preventing EGFR transport to the nucleus. There, EGFR would activate DNA-PK. DNA-PK is needed for the recruitment of DNA repair proteins and is involved in the non-homologous end-joining repair pathway [135,136]. Combinations of EGFR inhibitors with drugs that target DNA repair defective tumors showed increased cytotoxicity in HNSCC cell lines [135,137]. More recently, a similar therapeutic combination demonstrated enhanced radiation effects in HNSCC, both in vitro and in vivo [135].

The aurora kinase family is constituted of Aurora A, which plays a role in the regulation and stability of spindle assembly; Aurora B, essential for mitotic progression and cytokinesis; and Aurora C [138,139,140]. Aurora kinase A is commonly overexpressed in HNSCC, and coexpression with EGFR is associated with a poor prognosis [139]. EGFR can act as a transcriptional activator, leading to increased Aurora A gene expression [141]. Overexpression of Aurora B can induce tumor formation since it promotes chromosomal instability, and suppresses cell cycle inhibitor p21^Cip1^, and is associated with a poor outcome [142]. The combination of pan-aurora inhibitors with an EGFR mAb revealed additive inhibition of cell growth, even in cell lines resistant to Cetuximab, while a synergistic effect in ORL cell lines was reported for the combination with a TKI [138,143].

Double inhibition of the EGFR signaling pathway is a promising strategy to improve anticancer efficacy in tumors overexpressing EGFR. For instance, inhibition of HER-3 and EGFR in Cetuximab-resistant HNSCC cell lines and PDTX models enhanced the suppression of PI3K/AKT and ERK pathways and cell proliferation [106]. In a Cisplatin-resistant oral cancer cell line, a similar strategy showed synergistic proliferative inhibition, while in a cell line with higher expression of EGFR, a stronger cytotoxic effect and augmented pro-apoptotic activity were observed [144,145]. In clinical trials, this combination was usually well tolerated and had good anticancer activity, supporting preclinical findings [130,146]. However, the addition of Patritumab did not improve combinatorial Cetuximab and platinum therapy [147]. Thus, it is suggested that other pathways may be involved in the mechanism of resistance to Cetuximab treatment in HNSCC.

MAPK pathway signaling reactivation can occur in tumor cells after prolonged inhibition of EGFR, leading to the phosphorylation of ERK1/2. In OSCC tumor xenografts, inhibition of EGFR and ERK synergistically enhanced suppression of these pathways [148]. Nonetheless, a phase 1b study assessing the safety and tolerability of an identical approach showed generally poor tolerability, with no synergistic effects and limited efficacy [149].

Cancer cells can also escape EGFR inhibition through c-Met pathway activation [150]. c-Met is a membrane RTK involved in lymph node metastasis and drug resistance [5]. c-Met overexpression has been described in various HNSCC tumors and is associated with a poor prognosis [151]. EGFR signaling can activate c-Met and, in turn, c-Met can activate HER-3 signaling. Additionally, inhibition of one EGFR family member can lead to increased expression of the other members [5]. The inhibition of both EGFR and c-Met was shown to enhance anticancer efficacy in both cell lines and PDTX models [5]. Since the c-Met inhibitors had various targets, therapeutic effects could not be attributed to the inhibition of EGFR and ERK pathways. However, a randomized phase 2 clinical trial with patients with R/M HNSCC presented discouraging results, with no improvement in tumor response or OS and increased toxicity [152]. Nevertheless, it is suggested that future trials focusing on Met-aberration should include HNSCC patients, while Met inhibitors with higher potency and selectivity should continue to be explored.

Vascular endothelial growth factor receptor (VEGFR) is a receptor tyrosine kinase implicated in angiogenesis. Its silencing leads to inhibition of VEGF expression, while VEGF overexpression can lead to resistance to Cetuximab [153,154]. Targeting both EGFR and VEGF leads to growth delay and inhibition of tumor angiogenesis, both in vitro and in PDTX models [155]. In clinical trials, the combination of Cetuximab with Bevacizumab showed an increase in the disease control rate (DCR) and median OS when compared to platinum-based chemotherapy plus Cetuximab [155,156]. However, the increase in the response rate was small when compared with Cetuximab monotherapy (16% vs. 10–13%, respectively) [157]. Furthermore, Cetuximab plus Pazopanib in R/M HNSCC patients was deemed safe, and the preliminary data showed 47% of the patients achieving a tumor response [158]. However, Cetuximab plus Sorafenib exhibited higher toxicity than Cetuximab alone, and no significant survival difference was observed [153].

The PI3K/AKT/mTOR pathway is a well-described mechanism of resistance for Cetuximab, while increased levels of the transcription factor hypoxia-inducible factor 1 alpha (HIF-1α) are associated with resistance to Bevacizumab treatment [108,159]. Temsirolimus is an mTOR inhibitor that also regulates HIF-1α levels [160]. Thus, the addition of Temsirolimus should help overcome resistance to both drugs. However, in patients with advanced malignancies, this approach showed high toxicity, with 52% of patients developing grade 3 to 4 toxicities. The overall response rate, DCR, and PFS for the eight HNSCC patients were 25%, 38%, and 3 months, respectively [161]. Moreover, clinical trials exploring the addition of mTOR inhibitors to EGFR inhibitors have shown no significant benefit, even though these combinations were considered safe [162,163].

Bevacizumab was also combined with platinum-based chemotherapy in R/M HNSCC patients improving PFS and ORR, but not the OS [164]. Furthermore, increased toxicity was observed. The addition of radiation therapy to a similar therapeutic combination increased toxicity, although to acceptable levels, while showing promising efficacy results [165]. Adding Cetuximab to this combination led to a 2-year PFS of 88.5% [166]. Nonetheless, these results were probably influenced by patients’ characteristics associated with favorable prognosis in oropharynx cancer trials.

Cancer cells can counter EGFR inhibition through the activation of Src family kinases [167]. The synergistic activity of combined inhibition of Src and EGFR has been reported in several types of cancer [168,169]. In clinical trials, Dasatinib, an Src inhibitor, combined with EGFR inhibitors showed no clinical benefits, except for patients with low serum interleukin 6 (IL-6) levels. IL-6 can bypass SFK pathway inhibition and lead to STAT3 activation [170,171]. However, the association between low IL-6 serum levels and improved clinical benefits seems to be specific to the Dasatinib and Cetuximab combination.

Nuclear factor κB (NF-κB) is involved in pro-tumoral activity, EGFR signal transduction, and can lead to Gefitinib resistance. Accordingly, the addition of an NF-κB inhibitor resensitized OSCCs to Gefitinib [172].

The insulin-like growth factor (IGF) axis is comprised of the IGF type 1 receptor (IGF-1R), its ligands IGF-1 and IGF-2, and other proteins. It is involved in the regulation of cell proliferation, survival, metastization, and differentiation [173]. In addition, it is a known mechanism of resistance to Cetuximab therapy [174]. However, the addition of an IGF-1R inhibitor to Cetuximab in platinum-refractory HNSCC patients reported no PFS or OS improvements. The lack of efficacy may possibly be explained by existing redundant pathways that can overcome IGF-1R inhibition [175].

Other explored strategies involve the enhancement of Cetuximab activity. For instance, combination of Paclitaxel and Cetuximab has shown synergistic effects in HNSCC. Paclitaxel enhances antibody-dependent cellular cytotoxicity (ADCC), potentiating Cetuximab anticancer activity [176]. ADCC caused by Cetuximab is prompted when the antibody bound to a target cell also binds to any Fc-γ receptor of natural killer (NK) cells [177]. This leads to cell death through activation of tumor necrosis factor (TNF) death receptors and the release of cytotoxic granules and pro-inflammatory cytokines [178]. Still, the mechanism of potentiation of ADCC by Paclitaxel needs to be elucidated [176]. Similarly, the addition of Tipifarnib, a farnesyltransferase inhibitor (FTI), to Cetuximab in non-HRas mutated HNSCC showed better inhibitory activity than either drug alone [179]. Ras activation leads to enhanced gene transcription and fast cell proliferation [180]. Farnesylation is a crucial step in Ras activation, and FTIs directly interfere with this process [179]. However, the Ras isoforms K-Ras and N-Ras can overcome inhibition through an alternative process called geranylgeranylation, and, consequently, this combination will be more suitable to H-Ras HNSCC [181].

Treatment with the combination of a taxane with Trastuzumab has shown satisfactory results in HER-2-positive breast cancer [182]. In a case study report with patients with HER-2-positive salivary duct carcinoma (SDC), the combination showed an improvement in patients’ outcomes [183]. In addition, in a phase 2 trial, the combination of Docetaxel and Trastuzumab achieved an ORR of 70.2%, with an acceptable toxicity profile for HER-2-positive SDC patients [184]. Nevertheless, the results observed cannot be attributed to the combination until a study assessing Docetaxel as monotherapy in LA or R/M HER-2-positive SDC is conducted. Afatinib, a pan-EGFR inhibitor, was also combined with Docetaxel and postoperative radiation therapy, but high toxicity was observed [185]. Concurrent administration of Panitumumab, an anti-EGFR mAb, and Paclitaxel reported an ORR of 48%, a median OS of 9.9 months, and a median PFS of 7.5 months [186]. More recently, the same regimen as IC followed by radiotherapy and Panitumumab in patients with LA-HNSCC unfit for regimens with platinum drugs achieved a complete response in 8 patients and a partial response in 26. Nonetheless, the safety of this regimen was worse than anticipated, and further investigation was suggested [187].

In previous studies, Cetuximab in combination with a platinum and 5-FU exhibited a better response in OSCC patients [188]. Thus, addition of Cetuximab to the TPF regimen was explored, and an ORR of 88.4% was achieved. However, the OS did not improve. The regimen was considered effective, tolerable, and especially beneficial for patients with borderline inoperable cancers in advanced stages or tumors that have not spread to lymph nodes [189]. Although TPF IC is a prevalent treatment option in HNSCC, relapse of the disease is a common reason for the failure of the treatment [190]. Hence, other options have been investigated. A retrospective comparative study showed the addition of Cetuximab to a similar regimen showed no therapeutic benefit; however, the sample size limited the analysis [190]. On the other hand, Cetuximab with weekly Paclitaxel in R/M HNSCC was well tolerated and can be an option for patients that cannot receive platinum-based therapies [191]. Moreover, adding Carboplatin to this regimen obtained an ORR of 40%, a median OS of 14.7 months, and a PFS of 5.2 months, with acceptable toxicity. Still, a comparison with a platinum, 5-FU, and Cetuximab regimen should be investigated [192]. This same combination as IC followed by chemoradiotherapy with Cisplatin in LA-HNSCC showed considerable efficacy and a promising survival rate and is a good alternative to TPF IC [193]. The addition of Cisplatin to Cetuximab and Paclitaxel in R/M HNSCC reported a median OS comparable to the EXTREME regimen with reduced toxicity. A similar approach led to an ORR, OS, and PFS of 62%, 11.0 months, and 6.1 months, respectively [194]. Gefitinib enhanced the Cisplatin response in OSCC in vitro, while Cetuximab plus Cisplatin showed similar results to the combination with Paclitaxel, with a similar PFS, and can be used as a first-line treatment in patients with R/M HNSCC [195,196,197]. However, a regimen composed of Cetuximab, Docetaxel, and Cisplatin, when compared to the EXTREME treatment, showed no significant improvement in OS, even though this combinatorial approach can be an option for first-line treatment of R/M HNSCC patients, particularly those who cannot be treated with Pembrolizumab [198]. Combining Methotrexate, a dihydrofolate reductase inhibitor that hinders DNA synthesis, with Cetuximab led to higher PFS and clinical benefit rates than Methotrexate monotherapy in R/M HNSCC patients [199,200]. The combination of EGFR inhibitors and chemoradiotherapy with Cisplatin has been evaluated in both LA-HNSCC and R/M HNSCC. Two clinical trials were terminated due to the small number of patients and discouraging results from other trials with similar combinations, but the combination with Nimotuzumab was considered safe, with improved PFS and disease-free survival [115,201,202,203].

Cetuximab combined with radiotherapy is used for the treatment of LA-HNSCC; however, Cetuximab leads to an increase of cytotoxic T-lymphocyte-associated protein 4 (CTLA-4)-positive T-regulatory cells [204]. CTLA-4 is involved in the regulation of T cells, repressing their activation. The addition of a CTLA-4 inhibitor to Cetuximab plus radiotherapy in LA-HNSCC patients was tolerable and showed acceptable clinical activity, but did not meet the PFS endpoint [116].

Histone deacetylase (HDAC) inhibition can radiosensitize tumor cells, and a combination with a radiotherapy approach should lead to enhanced anticancer activity [205]. This regimen combined with EGFR or HER-2 inhibitors was deemed feasible for intermediate-/high-risk patients with HNSCC, but alternate schedules or routes of administration of the HDAC inhibitor are recommended for future trials due to five patients discontinuing its administration [206].

Strategies with EGFR inhibitors can successfully counter drug resistance and improve treatment efficacy. However, clinical trial results are usually ambiguous, with similar approaches showing both promising and discouraging results. This may demonstrate that combinatorial approaches should still be investigated with different drugs, even when no improvements are observed.

**Table 3:** Main combination therapies based on EGFR targeting used in clinical and preclinical trials for oral cancer treatment.

Combination Therapies	Study Design/ Treatment Type	Cancer Type and Stage	Main Reported Outcome	References
EGFR inhibition combined with radiotherapy	Phase 3 clinical trial -	LA-HNSCC III/IV	Exerted supra-additive effects, increasing OS.	[121]
	Retrospective study 1st and 2nd lines	OCSCC ≥I	Slightly less effective than when assessed for HNSCC patients, but deemed safe and with good efficacy.	[122]
Combination of EGFR and PI3K inhibitors	Phase 2 clinical trial 2nd and 3rd lines	R/M HNSCC II/III/IV	Exhibited no improvement of PFS, ORR, and OS.	[128]
	Phase 1 clinical trial Most patients previously treated	R/M HNSCC -	Associated with high toxicity and poor efficacy.	[129]
Alpelisib combined with Cetuximab and IMRT	Phase 1b clinical trial -	LA-HNSCC III/IVa/IVb	This treatment modality was considered safe.	[131]
Combination of EGFR inhibitors with drugs that target DNA repair defective tumors	Preclinical trial	N/A	Improved cytotoxicity and increased in vitro and in vivo radiation effects.	[135]
EGFR mAb combined with pan-aurora inhibitors	Preclinical trial	N/A	Exerted additive effects in inhibiting cell growth.	[138]
Combination of HER-3 and EGFR inhibitors	Preclinical trial	N/A	Enhanced the suppression of cell proliferation in vitro and in vivo.	[106]
	Phase 1/1b clinical trials Most patients previously treated	R/M HNSCC -	Showed great anticancer activity and was well tolerated.	[130,146]
Patritumab combined with Cetuximab and platinum	Phase 2 clinical trial 1st line	R/M HNSCC III/IVa-c	Exhibited good tolerability, but did not improve combination of Cetuximab and platinum.	[147]
Combination of EGFR and ERK inhibitors	Preclinical trial	N/A	Exerted synergistic effects, enhancing anticancer activity.	[148]
	Phase 1b clinical trial Patients previously treated	SGC -	Exerted no synergistic effects with limited efficacy and poor tolerability.	[149]
Combination of EGFR and c-Met inhibitors	Preclinical trial	N/A	Enhanced anticancer efficacy in in vitro and in vivo models.	[5]
	Phase 2 clinical trial ≥1st line	R/M HNSCC -	Displayed high toxicity associated with no improvement in tumor response or OS.	[152]
Combination of EGFR and VEGF inhibitors	Preclinical and phase 2 clinical trial Patients previously treated	R/M HNSCC -	Delayed tumor growth and inhibited tumor angiogenesis in vitro and in vivo. Deemed safe and showed activity in previously treated patients.	[155]
Cetuximab combined with Pazopanib	Phase 1b clinical trial ≥1st line	R/M HNSCC -	Showed safety and a good antitumor response.	[158]
Cetuximab combined with Sorafenib	Phase 2 clinical trial -	R/M HNSCC -	Exerted no significant survival response and showed high toxicity when compared to Cetuximab monotherapy.	[153]
Combination of Cetuximab, Bevacizumab, and Temsirolimus	Phase 1 clinical trial Mostly patients previously treated	HNSCC -	Exhibited good anticancer response; however, high toxicity was observed.	[161]
Combined Cetuximab and Temsirolimus	Phase 2 clinical trial R/M HNSCC	- -	Did not improve PFS, but induced response in Cetuximab refractory patients with good safety profile.	[162]
Everolimus in combination with Erlotinib	Phase 2 clinical trial Patients previously treated and untreated	R/M HNSCC -	Was deemed safe, but no benefit was observed for this combination.	[163]
Bevacizumab combined with platinum-based chemotherapy	Phase 3 clinical trial Patients previously treated and untreated	R/M HNSCC -	Exhibited no significant OS improvement and increased toxicity. Nonetheless, PFS and ORR improved.	[164]
Bevacizumab in combination with Cisplatin and IMRT	Phase 2 clinical trial 1st line	LA-HNSCC III/IVa/IVb	Slightly increased toxicity and promising efficacy were observed.	[165]
Combination of Bevacizumab, Cetuximab, Cisplatin, and IMRT	Phase 2 clinical trial -	LA-HNSCC III/IVa/IVb	Exhibited good tolerability and anticancer activity.	[166]
Dasatinib combined with EGFR inhibitor	Clinical trial -	HNSCC I–IV	Showed no clinical benefits.	[170]
	Phase II clinical trial -	R/M HNSCC -	Showed clinical relevance in patients with low serum IL-6 levels.	[171]
Combination of anti-IGF1R with Cetuximab	Phase 2 clinical trial -	R/M HNSCC -	Exhibited no significant improvement of OS and PFS.	[175]
Combination of Paclitaxel and Cetuximab	Preclinical trial	N/A	Exerted synergistic effects, increasing anticancer response.	[176]
	Phase 2 clinical trial 1st line	R/M HNSCC -	Exhibited antitumoral activity and good tolerability.	[191]
Combination of Tipifarnib and Cetuximab	Preclinical trial	N/A	Enhanced cell inhibitory activity.	[179]
Combination of Docetaxel and Trastuzumab	Phase 2 clinical trial Patients previously treated	SDC -	Showed an acceptable toxicity profile and promising efficacy for HER-2-positive SDC patients	[184]
Afatinib combined with Docetaxel and postoperative radiation therapy	Phase 1 clinical trial -	LA-HNSCC II/III/IV	Exhibited high toxicity.	[185]
Panitumumab in combination with Paclitaxel	Phase 2 clinical trial Patients previously treated	R/M HNSCC -	Exhibited good anticancer activity and was considered safe.	[186]
Panitumumab combined with Paclitaxel followed by radiotherapy and Panitumumab	Phase 2 clinical trial 1st line (IC)	LA-HNSCC III/IVa/IVb	Toxicity worse than expected; however, led to a higher ORR.	[187]
Cetuximab combined with a platinum and 5-FU	Phase 3 clinical trial 1st line	R/M HNSCC -	Displayed good anticancer response in OSCC patients, improving OS.	[188]
Combination of Cetuximab with TPF	Phase 2 clinical trial 1st line (IC)	LA OCSCC IV	Exhibited a high ORR, was well tolerated and effective; however, no significant improvement of OS was observed.	[189]
Combination of Cetuximab, Paclitaxel, and Carboplatin	Phase 2 clinical trial 1st line	R/M HNSCC III/IV	Showed good anticancer activity with acceptable toxicity.	[192]
	Phase 2 clinical trial 1st line (IC)	LA-HNSCC IVa/IVb	Good anticancer activity and promising survival was observed.	[193]
Combination of Cetuximab, Paclitaxel, and Cisplatin	Phase 2 clinical trial 1st line	R/M HNSCC -	Exhibited a moderate OS and low toxicity.	[194]
	Phase 2 clinical trial 1st line	R/M HNSCC -	No significant change in OS with the addition of Paclitaxel.	[195]
	Phase 2b clinical trial 1st line	R/M HNSCC -	Addition of Paclitaxel did not improve patient’s outcome.	[196]
Gefitinib combined with Cisplatin	Preclinical trial	N/A	Exacerbated in vitro anticancer activity.	[197]
Combination of Cetuximab, Docetaxel, and Cisplatin	Phase 2 clinical trial 1st line	R/M HNSCC -	Exhibited no significant improvement of OS when compared to EXTREME regime. However, can be an alternative in first-line treatment.	[198]
Methotrexate combined with Cetuximab	Phase 1b/2 clinical trial 1st line	R/M HNSCC -	Improved PFS and clinical efficacy.	[199]
	Retrospective study ≥1st line	R/M HNSCC -	The treatment was deemed safe and is an option for palliative treatment.	[200]
Concurrent radiotherapy, Dacomitinib, and Cisplatin	Phase 1 clinical trial -	LA-HNSCC III/IVa/IVb	Tolerable side effects, but the study was early terminated since other studies showed high toxicity profiles and no improvement in the outcomes.	[201]
Vandetanib combined with Cisplatin and radiotherapy	Phase 1 clinical trial 1st line	LA-HNSCC III/IV	Well tolerated.	[202]
Combination of Nimotuzumab with Cisplatin and radiotherapy	Phase 3 clinical trial -	LA-HNSCC III/IV	Improved PFS and DFS.	[203]
	Phase 2 clinical trial 1st line	LA-HNSCC III/IV	Exhibited good tolerability, with high response rates.	[115]
Addition of Ipilimumab to concurrent Cetuximab and radiotherapy	Phase 1 clinical trial 1st line	LA-HNSCC III/IVa/IVb	Well tolerated and showed clinical activity. However, it did not meet PFS endpoint.	[116]
Combination of histone deacetylase inhibitor with radiotherapy and EGFR or HER-2 inhibitors	Phase 1 clinical trial -	LA-HNSCC III/IV	Exhibited good tolerability at biologically effective doses.	[206]

Abbreviations: DFS, disease-free survival; EGFR, epidermal growth factor receptor; HER-2, human epidermal growth factor receptor 2; HNSCC, head and neck squamous cell carcinoma; IC; induction chemotherapy; LA-HNSCC, locally advanced head and neck squamous cell carcinoma; LA OCSCC, locally advanced oral cavity squamous cell carcinoma; N/A, not applicable; ORR, objective response rate; OS, overall survival; PFS, progression-free survival; R/M HNSCC, recurrent/metastatic head and neck squamous cell carcinoma; SDC, salivary duct carcinoma; VEGF, vascular endothelial growth factor; -, data not provided.

### 3.3. Cyclin-Dependent Kinase Inhibition-Based Combination Therapies

**Figure 3:**
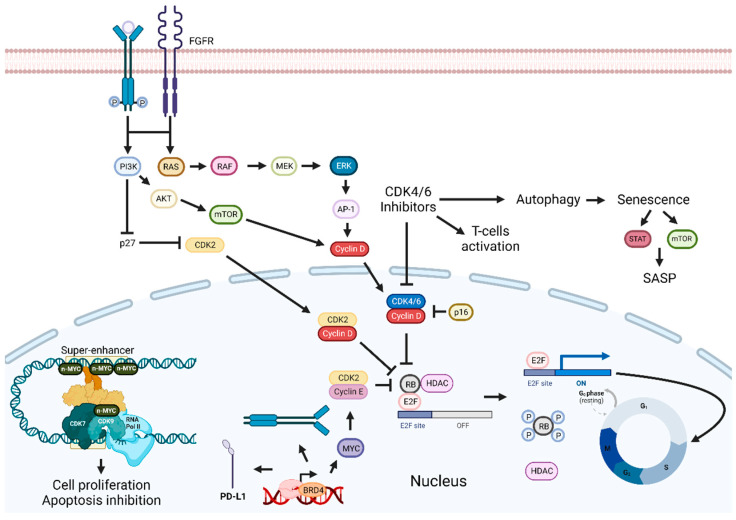
Cyclin-dependent kinases and BET inhibition enhancement pathways. Inhibition of the p16-Cyclin D1-CDK4/6-Rb pathway leads to cell cycle arrest and tumor growth suppression. Combining CDKIs with FGFR, EGFR/ERK inhibitors, senolytic drugs, HDAC, BET, or PI3K inhibitors can enhance therapeutic outcomes. Targeting BET proteins hinders cancer development. Combinatorial approaches with BET inhibitors and PI3K inhibitors, drugs targeting proteins involved in transcription or immune checkpoint inhibitors, should improve antitumoral effects. Abbreviations: AP-1, Activator protein 1; AKT, BET, bromodomain and extra-terminal domain; BRD4, bromodomain-containing protein 4; CDK, cyclin-dependent kinase; CDKIs, CDK inhibitors; EGFR, epidermal growth factor receptor; ERK, extracellular signal-regulated kinases; E2F, transcription factor; FGFR, fibroblast-growth-factor receptor; G0, Gap 0; G1, Gap 1; HDAC, histone deacetylase; MEK, MAPK/ERK kinase; M, mitosis; mTOR, mammalian target of rapamycin; PD-L1, programmed death-ligand 1; Rb, retinoblastoma protein; RNA pol II; RNA polymerase II; S, synthesis phase; SASP, senescence-associated secretory phenotype. Created by the authors with BioRender.com.

Cancer is characterized by increased proliferation, and cyclin-dependent kinases (CDKs) are essential in this process [207]. CDKs control the cell cycle, and in tumors, the p16-Cyclin D1-CDK4/6-Rb pathway is usually dysregulated [208]. These protein kinases are a part of the serine/threonine subfamily and are functionally divided into two subgroups: one implicated in the cell cycle (CDK1, CDK2, CDK4, and CDK6) and the other in transcription (CDK7, CDK8, and CDK9) [207,209]. CDK2 regulates the cell cycle through the S phase, while the transition from G2 to the M phase is regulated by CDK1. CDK7 is associated with transcription initiation and can also activate other CDKs through phosphorylation [210]. CDK4 and CDK6, along with cyclin D1, control the transition from G1 to the S phase by regulating the phosphorylation state of Rb [211]. Rb blocks the G1/S transition, but when it becomes phosphorylated, its activity is inhibited, and the cell cycle can proceed (Figure 3) [211].

Treatment that inhibits the complex formed by CDK4/6 and cyclin D1 should lead to cell cycle arrest and tumor growth inhibition. However, Palbociclib, a CDK4/6 inhibitor, leads to the induction of mTOR1/S6 phosphorylation, which increases cell metabolism and ATP generation, a probable mechanism of resistance to this therapy [208]. Afatinib inhibits EGFR/ERK signaling and reduces cyclin D1 expression, leading to cell cycle arrest, and can potentially inhibit the development of resistance to Palbociclib. Consequently, Palbociclib and Afatinib promoted cell cycle arrest, induced cellular senescence, and inhibited tumor growth [208]. A similar therapeutic approach using Ribociclib and Cetuximab in some HPV-negative HNSCC PDTX models improved slightly or had the same response as these drugs administered as monotherapies [212]. However, in the Cetuximab-resistant HNSCC PDTX model, this combination was less effective than Ribociclib alone. Most likely due to the reduction in Rb expression in the resistant model, which is supported by the existence of a positive correlation between Ribociclib activity and Rb expression levels [213]. Published results from clinical trials exploring this approach show mixed outcomes. For example, two studies reported anticancer activity and tolerable adverse reactions, while a phase 2 trial with patients with platinum-resistant, Cetuximab-naïve, HPV-unrelated HNSCC showed no significant improvement in OS and increased adverse events when compared to Cetuximab alone [214,215,216]. Notably, the median OS for Cetuximab monotherapy was higher than reported in other trials, probably because this was the only trial with exclusively HPV-unrelated HNSCC patients. Nevertheless, an improvement in OS was found in patients with tumors expressing lower levels of cyclin D1, with no *PIK3CA* mutations or CDK4/6 amplification. In addition, in a phase 2 trial with Cetuximab-resistant, HPV-related oropharynx squamous cell carcinoma patients, minimal anticancer activity and an ORR of only 4% were reported [217].

Rb is involved in senescence, and, thus, CDK4/6 inhibition leads to a cellular senescence phenotype [217]. Cells with this phenotype produce and secrete chemokines, cytokines, and several other bioactive molecules that constitute the senescence-associated secretory phenotype (SASP) [218]. SASP is involved in both the inhibition and promotion of cancer. To improve the efficacy of CDK4/6 inhibitors, a combination with a senolytic or a senostatic drug could be explored. Senolytic drugs induce apoptosis in senescent cells, while senostatic ones repress a senescent phenotype, without promoting apoptosis [218,219,220]. The latter combination led to inhibition of the mTOR and STAT3 pathways in senescent HNSCC, and consequent suppression of SASP-induced stemness [218].

Cyclin D1 and p16 are commonly deregulated in NPC. Rb mutations are infrequent, making the blockade of CDK4/6 a promising therapy. The combination of a CDK4/6 inhibitor with Suberanilohydroxamic acid, an HDAC inhibitor, led to enhanced suppression of NPC cell growth, both in vitro and in vivo, possibly by potentiating the autophagy-inducing effect of Palbociclib. In contrast, when Palbociclib was combined with Cisplatin, an antagonistic effect was observed, possibly due to the inhibitory effects of Palbociclib on the cell cycle, interfering with the cytotoxic effect of Cisplatin [218]. A similar approach with Carboplatin for unresectable R/M HNSCC presented unsatisfactory antitumor activity and high toxicity [219,220]. These results could be explained by the fact that most patients were HPV-negative, which is associated with a worse response to therapy, and by the induction of chemotherapy-resistant G0 dormancy [220].

Resistance to Palbociclib therapy has been identified in Cisplatin-resistant HNSCC, as cyclin E is overexpressed and CDK2 is hyperactivated in resistant cell lines [221]. Cyclin E and CDK2 are involved in the inactivation of Rb and in the promotion of cell cycle progression, overcoming Palbociclib activity [222]. To bypass Palbociclib resistance, the use of JQ1 was proposed. JQ1 is a BRD4 inhibitor that consequently downregulates c-Myc, leading to Rb dephosphorylation. This combination showed synergistic effects, causing a significant reduction in tumor volume two weeks after administration in vivo, suggesting it could be an interesting treatment option for Cisplatin-resistant HNSCC [221]. Cancer cells with mutations in *PIK3CA* are resistant to CDK4/6 inhibitors, possibly due to cyclin D1 and CDK2 pathway activation. The combination with a PI3K inhibitor reduced cyclin D1 and CDK2 expression and accumulation of Rb non-phosphorylated in vitro, while showing controlled tumor growth both in vitro and in vivo [223]. In tonsillar squamous cell carcinoma (TSCC) cell lines, a similar combination showed synergistic effects [224].

Overexpression of fibroblast-growth-factor receptor (FGFR) 3 has been associated with a poor prognosis in TSCC, and recently, FGFR inhibitors were approved for the treatment of breast cancer [224,225]. Thus, the combination of CDK 4/6 inhibitors with FGFR inhibitors was assessed and showed different effects, with some combinations presenting synergistic activity. Previously, a FGFR inhibitor was combined with a PI3K inhibitor, leading to inhibition of TSCC and the base of tongue squamous cell carcinoma cells growth and increased antitumor effects [225].

In addition to the therapeutic combination and dose of each drug, the sequential timing of each combinatorial drug is also important for treatment efficacy [207]. For instance, a CDK4/6 inhibitor is expected to have a favorable outcome when applied first since it helps preserve hematopoietic stem and progenitor cells through the induction of G1 arrest and enhanced antitumor immunity, as observed with Palbociclib [207,226]. In a recent study, dual-CDK inhibition presented a synergistic effect, while therapeutic results for sequential application of this combination were dependent on the inhibitor used first. THZ1 (a selective CDK7 inhibitor) combined with 5-FU reduced impedance and led to a G1 cell cycle arrest. Dinaciclib and THZ1 with 5-FU treatments induced CalR translocation and upregulation of MHC class I, making them promising drugs for immunotherapeutic strategies. Dinaciclib and Cisplatin were tested in vivo and improved growth suppression.

Tumor cells can develop resistance to radiation therapy; thus, approaches to overcome resistance have been explored. For instance, addition of Palbociclib in OCSCC, enhanced radiation sensitivity by disrupting DNA repair pathways [227].

Concurrent therapeutic strategies with CDK inhibitors showed good results in preclinical trials, with improvements in anticancer effects and in overcoming resistance. Nonetheless, clinical trials exploring some of these combinations usually report high toxicity, leaving these drugs far from clinical use (Table 4).

### 3.4. Bromodomain and Extra-Terminal Domain (BET) Proteins Inhibition-Based Combination Therapies

Bromodomain and extra-terminal domain (BET) proteins play a role in the control of genome activity and in the regulation of transcription in cellular differentiation [228,229]. BRD4 is a BET family member that acts as a transcriptional and epigenetic regulator, is involved in cancer development, and is often overexpressed in OSCCs [228,230]. Since oncogenes seem to be dependent on BRD4, this protein arises as a possible good therapeutic target (Figure 3) [230]. JQ1 has been one of the most explored BRD4 inhibitors; however, cancer cells can acquire resistance to this drug through the activation of the EGFR pathway [231]. A combinatorial approach with JQ1 and a PI3K inhibitor showed synergistic effects in the treatment of several types of cancer cells [228,232]. In OSCCs, this combination increased the efficacy of the treatment, both in vivo and in vitro, by enhancing the suppression of EGFR and BRD4 expression [228]. Enhancer remodeling, transcriptional plasticity, and heterogeneity are key factors that also confer cancer cells the ability to develop resistance to BRD4 inhibitors [233,234]. BRD4 was also shown to play a major role in RNA polymerase II (RNAPII) activation and in modulating super-enhancer (SE)-associated genes [235]. SE is a cluster of enhancers involved in the regulation of cell-specific genes essential for cellular identity [236]. CDK7 regulates RNAPII action by regulating its phosphorylation status. It is also involved in the regulation of transcription, mainly regulating SE-associated genes [235]. These genes are associated with cancer development. Targeting both BRD4 and CDK7 in OSCCs led to synergistic anticancer effects, both in vivo and in vitro, through modulation of a SE-associated gene known as Yes-associated protein (YAP) 1, involved in the promotion of cell proliferation and apoptosis inhibition [235]. In HNSCC cancer stem cells, the combination of a YAP inhibitor with melatonin led to synergistic effects, with increased apoptosis and mitochondrial impairment, while reducing metastasis formation [237]. BET inhibitors, including JQ1, have also been shown to reduce Forkhead box M1 (FOXM1) expression in some tumors [238,239]. FOXM1 is a transcription factor associated with DNA damage repair, proliferation, and metastasis [240]. JQ1 can also inhibit programmed cell death-ligand 1 (PD-L1) expression [239]. PD-L1 is linked to therapeutic resistance and inhibition of anticancer immunity by suppressing the activation of T cells [239,241]. A combination of JQ1 and small interfering RNA targeting PD-L1 increased growth inhibition in vitro and in vivo through reduction of the expression of BRD4, c-MYC, FOXM1, and PD-L1 [239].

Combinatorial approaches with BET inhibitors have been mostly focused on JQ1 to improve effectiveness and overcome resistance, and with some success. However, we found no clinical trials results and no undergoing trials with BET inhibitors in HNSCC (Table 5).

### 3.5. PD-1 and PD-L1 Inhibition-Based Combination Therapies

**Figure 4:**
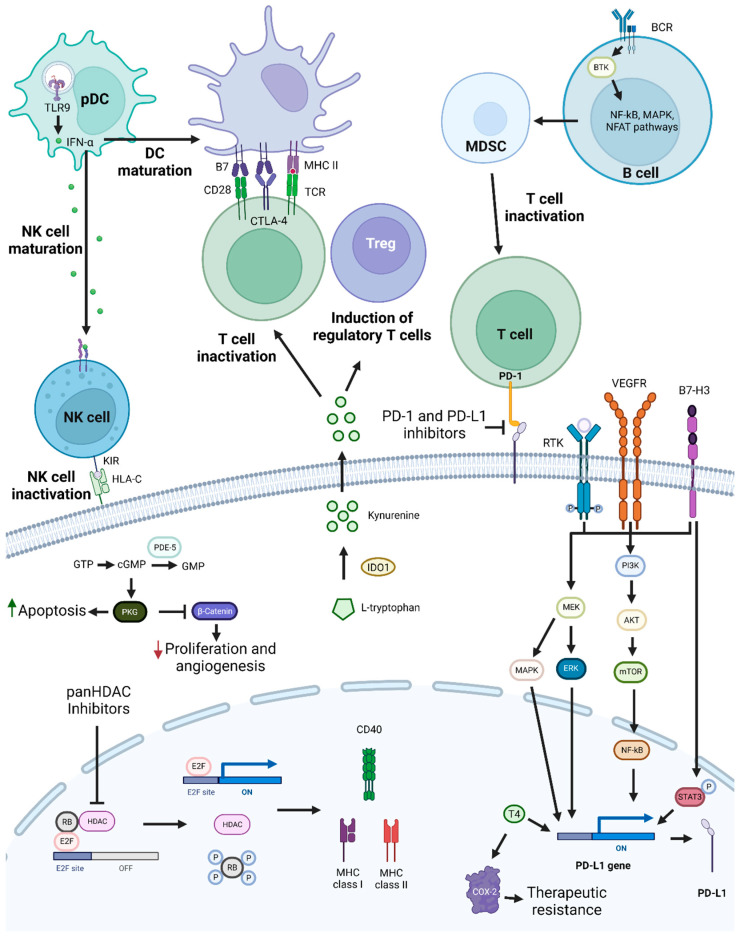
PD-1/PD-L1 targeting and possible synergistic co-targeted pathways. PD-1 and PD-L1 targeting leads to T-cell activation, which increases antitumoral immune responses. To achieve synergistic effects, combinations of PD-1/PD-L1 inhibitors with TLR9 agonists, HDAC inhibitors, BTK, KIR, PDE-5 inhibitors, T4 inhibitors, drugs targeting molecules involved in T-cell activation repression, or drugs targeting pathways and proteins involved in tumor cell growth and survival mechanisms, such as PI3K/AKT, MEK, and STAT3, may be used. Abbreviations: B7-H3, B7 homolog 3 protein; BCR, B-cell receptor; BTK, Bruton’s tyrosine kinase; cGMP, cyclic guanosine monophosphate; COX-2, cyclooxygenase-2; CTLA-4, cytotoxic T-lymphocyte-associated protein 4; GMP, guanosine monophosphate; GTP, guanosine triphosphate; HDAC, histone deacetylase; HLA-C, human leukocyte antigen-C; IDO1, Indoleamine 2,3-Dioxygenase 1; IFN-α, interferon alfa; KIR, killer-cell immunoglobulin-like receptor; MAPK, mitogen-activated protein kinases; MDSC, myeloid-derived suppressor cells; MHC, major histocompatibility complex; NF-κB, nuclear factor κB; NFAT, nuclear factor of activated T cells; NK, natural killer; pDC, plasmacytoid dendritic cell; PDE-5, phosphodiesterase-5; PD-L1, programmed death-ligand 1; PKG, cyclic GMP-dependent protein kinase; Rb, retinoblastoma protein; RTK, receptor tyrosine kinase; T4, thyroxine; TCR, toll-like receptor; TLR9, toll-like receptor 9; Treg, T regulatory cell; VEGFR, vascular endothelial growth factor receptor. Created by the authors with BioRender.com.

Immune checkpoints are crucial for regulating immune responses and preventing autoimmune reactions. However, in cancer, these checkpoints are often exploited to suppress the antitumor immune response. Consequently, checkpoint inhibitor antibodies have been developed and are currently undergoing clinical trials or have been approved for various cancer types [242]. Tumor cells have the ability to evade the immune system, as they can suppress immune responses by inducing immune checkpoint pathways [243]. PD-1 and PD-L1 proteins are involved in immune checkpoint activation and, by inhibiting T-cell activation, are crucial to maintain immune tolerance (Figure 4). However, the PD-1/PD-L1 axis is also responsible for cancer immune escape. Indeed, PD-L1 overexpression has been reported in various types of cancers and is associated with a poor prognosis [241].

Anti-PD-1 mAbs have recently been approved for the treatment of HNSCC. Thus, several therapeutic combinations targeting PD-1 and PD-L1 are being evaluated in ongoing clinical trials (NCT03082534, NCT04473716, NCT05063552) (Table 6). Nivolumab is one of the recently approved drugs targeting PD-1. Patients’ response to anti-PD1 therapies is reduced by an immunosuppressive tumor microenvironment. Combining Sitravatinib, an RTK inhibitor, with Nivolumab should inhibit the pathways involved in this process. In patients with OCC, a potential additive/synergistic effect of this combination was observed since 50% of the patients revealed viable tumor reduction, regardless of PD-L1 expression [244]. The regimen was safe, and downstaging from clinical to pathological happened in 90% of patients.

B7 homolog 3 protein (B7-H3) is a molecule of the B7 family associated with pro-tumorigenic activity and repression of T-cell activation. Concurrent inhibition of PD-1 and B7-H3 was considered safe and led to a 33% ORR in HNSCC patients [245]. Targeting both PD-1 and CTLA-4 in melanoma led to a higher objective and pathologic response [246]. In untreated OCSCC, this approach was deemed safe, and a pathologic downstaging of 69% and a pathologic response of 73% were described. It was also reported that 3 patients had a complete pathologic response greater than 90% [247]. In a phase 3 trial with R/M HNSCC patients, this combination as first-line therapy did not meet the endpoint for OS, but showed a better safety profile than the EXTREME regimen [248]. A similar approach with Durvalumab and Tremelimumab did not improve OS in patients with high expression of PD-L1. It also led to a lower median PFS, although grade 3/4 adverse events were higher for the EXTREME regimen. The similar OS observed was possibly due to subsequent immunotherapy in some patients of the EXTREME arm [249]. Comparably, the same combination with R/M HNSCC patients showed no OS improvement when compared to standard of care (Cetuximab, a taxane, Methotrexate, or a fluoropyrimidine) [250]. In a mouse model of pancreatic ductal adenocarcinoma, Bruton’s tyrosine kinase inhibition led to increased CD8+ antitumor activity [251]. Thus, a combination with an anti-PD-1 mAb was explored in HNSCC and showed addictive effects, although no clinical benefit was reported. Additionally high toxicity was observed [252]. The activation of toll-like-receptor (TLR) 9 by SD-101, a TLR9 agonist, increases immune responses by promoting the production of interferon alpha (IFN-α) and dendritic cells maturation into antigen-presenting cells. IFN-α consequently stimulates NK cells maturation [253]. In R/M HNSCC the combination of a TLR9 agonist with a PD-1 inhibitor did not induce clinically significant responses, even though objective responses were observed for 24% of the patients. Nonetheless, the treatment enhanced immune activity especially for patients benefiting with the combination [254]. Killer immunoglobulin-like receptors (KIR) are involved in the regulation of NK cells. Lirilumab is an anti-KIR2DL1, KIR2DL2, and KIR2DL3 mAb. These KIRs are involved in the negative regulation of NKs by binding to human leukocyte antigen C [255]. Thus, addition of Lirilumab should lead to the activation of NKs, which have an important role in innate immunity. A phase 2 trial exploring the concurrent administration of Lirilumab and a PD-1 inhibition showed good tolerability and a pathological response rate of 43%. A 2-year OS of 80% was also observed [256].

Targeting of proteins that prompt angiogenesis have been shown to promote a less immunosuppressive tumoral microenvironment; thus, combinations of anti-PD-1 mAbs with VEGFR inhibitors have been explored in clinical trials, showing the treatment was well tolerated, with promising antitumor activity [257,258]. Phosphodiesterase-5 (PDE-5) is overexpressed in various types of cancer and is associated with tumorigenesis through the downregulation of cyclic guanosine monophosphate (cGMP) [259,260,261,262]. cGMP has an important role in the regulation of cancer cell growth, angiogenesis, and anticancer immune activity [261,263]. Thus, inhibition of PDE-5 should lead to a less immunosuppressive tumor microenvironment. When combined with Nivolumab, it led to a pathological treatment response in 50% of the HNSCC patients. It also increased immune activity in the tumoral microenvironment. In addition, the treatment was considered safe [263].

PD-1 and PD-L1 inhibitors have also been investigated in combination with some of the most used drugs for the treatment of HNSCC. For instance, a combination of an anti-PD-1 drug with Docetaxel reached a median PFS of 5.8 months and a median OS of 21.3 months in R/M HNSCC patients and had a manageable toxicity profile [264]. In LA-HNSCC a similar combination plus platinum led to a high ORR and was deemed tolerable [265]. While an PD-1 inhibitor combined, as IC, with TPF, led to a better ORR and PFS than IC alone, with no increase in side effects [266]. Increased PD-L1 positive samples after IC and CD8+ lymphocytes infiltration are some of the suggested explanations for this outcome. However, the OS showed no improvement. Addition of Toripalimab, an anti-PD-1 mAb, to a combination of Cisplatin and gemcitabine was deemed safe and led to an increase of CD20 expression which correlates with the pathological response rate. This suggests that B cells might have a crucial role in anticancer immunity [267].

Pembrolizumab has also recently been approved for the treatment of HNSCC. A phase 3 clinical trial comparing a combination of Pembrolizumab with chemotherapy vs. Cetuximab with chemotherapy showed PFS, ORR, and adverse reactions were similar for both treatments. Nevertheless, a better and longer duration of response and improved OS were observed for the combination with Pembrolizumab [28]. These findings suggest that early exposure to Pembrolizumab can sensitize tumors to subsequent therapy. It was also shown that Pembrolizumab monotherapy can be used as a first-line treatment for PD-L1-positive R/M HNSCC. A 4-year follow-up showed a continued survival benefit of Pembrolizumab monotherapy and Pembrolizumab combined with chemotherapy over the combination of Cetuximab and chemotherapy [268]. In a phase 3 trial, the addition of Avelumab to chemoradiotherapy with Cisplatin did not improve PFS [269]. Avelumab and Cetuximab with radiotherapy in LA-HNSCC improved PFS but did not meet the endpoint [270]. The combination of Nivolumab and Cetuximab in R/M HNSCC patients was deemed safe with promising anticancer activity. Additionally higher ORR was observed for p16-negative patients [271]. The addition of Palbociclib to the combination of Avelumab and Cetuximab led to a good response and can be safely administered however the increased hematological toxicity with no clear outcome improvement when compared with combinations of Cetuximab and immune checkpoint inhibitors can restrain further investigation [272].

In a phase 2 study, the combination of Pembrolizumab and Cetuximab led to an ORR of 45%, which was higher than that of the individual drug treatments [273]. A median OS of 18.4 months and a slight increase in toxicity were observed. While a similar combination in a phase 2 study, with Afatinib plus Pembrolizumab, achieved a PFS of 4.1 months, and an OS of 8.9 months [274]. The addition of Cetuximab to Nivolumab was also investigated in R/M HNSCC patients, and, while deemed safe, no significant OS improvement was reported [275].

Synergistic activity has been observed for epigenetic modifications and PD-1 inhibition. Combined Pembrolizumab and vorinostat, a panHDAC inhibitor, for the treatment of R/M HNSCC and salivary gland cancer (SGC), demonstrated anticancer activity in both diseases, but with better results for HNSCC patients. For HNSCC patients, 8 (32%) partial responses were observed, while only 4 (16%) SGC patients showed the same response. The median OS was 12.6 months and the median PFS was 4.5 months for HNSCC, while in SGC the median OS was 14.0 months and the median PFS was 6.9 months [276].

Various pathways and molecules are involved in the expression of PD-L1 such as STAT3, PI3K, and thyroxine (T4) [277]. Inhibition of PD-L1 with resveratrol induces antiproliferative effects in vitro and in vivo [278]. The antiproliferative activity promoted by resveratrol occurs through the accumulation of COX-2 in the nucleus induced by the activation of ERK1/2 [277]. PD-L1 induced by T4 prevents the accumulation of nuclear COX-2, attenuating resveratrol activity [279]. Combination of resveratrol with a T4 inhibitor led to reduced PD-L1 expression and enhanced proliferation inhibition in oral cancer cell lines, probably by blocking PI3K-STAT3 signaling that consequently suppressed the inhibitory effect of pro-inflammatory genes induced by T4 [277]. In the same report, a combination of resveratrol and a STAT3 inhibitor showed a similar effect.

Different kinases such as EGFR, mTOR and ERK are involved in resistance to treatment with PD-1 and PD-L1 inhibitors. Thus, a combination with a multi-kinase inhibitor was tested in OSCC in vivo showing enhanced efficacy by inhibiting immunosuppressive cells while promoting anticancer immune cells activity [280]. MEK is described to increase expression of tumor antigens and PD-L1, thus, in a phase II trial a combination of an anti-PD-L1 mAb and a MEK inhibitor was explored leading to no activity for patients previously treated with anti-PD-1/PD-L1 drugs. Nonetheless, moderate activity was observed in HNSCC patients who did not receive previous treatment with those inhibitors [281].

Concurrent treatment of an indoleamine 2,3-Dioxygenase 1 (IDO1), an enzyme that catabolizes tryptophan, inhibitor with a PD-L1, or a PD-1 inhibitor improves the efficacy of checkpoint blockade [282]. IDO1 suppresses T-cell activity and hyperactivates regulatory T cells by depletion of L-tryptophan and accumulation of kynurenine. Therefore, IDO1 inhibition is not expected to directly kill cancer cells [283,284]. A combination of Epacadostat and Pembrolizumab was deemed safe, and one HNSCC patient achieved a partial response [285]. However, the results of a clinical trial, conducted with a similar combinatorial modality, revealed no difference when compared to atezolizumab alone [284].

With the approval of Pembrolizumab and Nivolumab for clinical use, most combinations undergoing clinical trials target PD-1 and PD-L1. The published clinical trials’ results indicate predominantly favorable outcomes for the combinations, indicating that further investigation into such combinational strategies should be pursued.

**Table 6:** Main combination therapies based on PD-1 and PDL-1 targeting used in clinical and preclinical trials for oral cancer treatment.

Combination Therapies	Study Design/Treatment Type	Cancer Type and Stage	Main Reported Outcome	References
Sitravatinib combined with Nivolumab	Clinical trial Preoperative	LA-HNSCC III/IVa	Exerted synergistic/addictive effects, promoting tumor reduction, and showed good safety.	[244]
Combination of PD-1 and B7-H3 inhibitors	Phase 1/2 clinical trial -	R/M HNSCC -	Showed good tolerance, acceptable safety profile, and antitumoral activity.	[245]
Combination of PD-1 and CTLA-4 inhibitors	Phase 2 clinical trial -	LA-OCSCC II/III/IVa	Exhibited high pathologic response and good safety profile.	[247]
	Phase 3 clinical trial 1st line	R/M HNSCC -	Better toxicity profile than the EXTREME regimen, but did not meet endpoint of OS.	[248]
	Phase 3 clinical trial 1st line	R/M HNSCC -	Did not improve OS in patients with high expression of PD-L1. Led to lower median PFS, but with less grade 3/4 adverse events.	[249]
	Phase 3 clinical trial 2nd line	R/M HNSCC -	Showed no significant improvement of OS comparing to standard therapies.	[250]
VEGFR inhibitors with anti-PD-1 mAbs	Phase 1 clinical trial Neoadjuvant	LA-OSCC III/IVa	Well tolerated and showed a major pathological response rate of 40%	[257]
	Phase 1b/2 clinical trial ≥1st line	R/M HNSCC -	Deemed tolerable and displayed promising anticancer activity.	[258]
Nivolumab in combination with Tadalafil	Clinical trial Neoadjuvant	HNSCC -	The treatment was safe, with 50% of the patients showing pathological treatment response.	[263]
Pembrolizumab plus Acalabrutinib	Phase 2 clinical trial -	R/M HNSCC -	Showed no clinical benefit with high toxicity.	[252]
Pembrolizumab combined with SD-101	Phase 2 clinical trial ≥1st line	R/M HNSCC -	Overall, 24% of the patients showed objective response but did not reach the threshold.	[254]
Combination of Nivolumab and Lirilumab	Phase 2 clinical trial Neoadjuvant/ adjuvant	LRR HNSCC I–IV	Led to high 2-year OS and a pathological response rate of 43%.	[256]
Pembrolizumab plus Docetaxel	Phase 1/2 clinical trial ≥1st line	R/M HNSCC -	Achieved a median PFS of 5.8 months and a median OS of 21.3 with manageable toxicity.	[264]
Camrelizumab, Paclitaxel or Docetaxel and Cisplatin	Phase 2 clinical trial Neoadjuvant	LA-HNSCC III/IVa/IVb	Led to high ORR and was well tolerated.	[265]
TPF combined with an anti-PD-1	Clinical trial 1st (IC)	LA-HNSCC III/IV	Displayed greater ORR and PFS than monotherapies, with no significant increase in adverse effects; however, no improvement of OS was observed.	[266]
Addition of Toripalimab to Gemcitabine and Cisplatin	Phase 1b clinical trial Neoadjuvant	LA-HNSCC III/IVa/IVb	The therapy was considered safe and led to an increase in CD20 expression.	[267]
Combination of Pembrolizumab with a platinum and 5-FU	Phase 3 clinical trial 1st line	R/M HNSCC -	Appropriate first-line treatment for R/M HNSCC. Four-year follow up showed continued survival benefit.	[28,268]
Addition of Avelumab to standard-of-care chemoradiotherapy	Phase 3 clinical trial -	LA-HNSCC III/IVa/IVb	The objective of prolonging PFS was not achieved.	[269]
Avelumab combined with Cetuximab and radiotherapy	Phase 2 clinical trial -	LA-HNSCC III/IV	Deemed tolerable. Improved PFS, but did not meet the endpoint	[270]
Nivolumab in combination with Cetuximab	Phase 2 clinical trial Patients previously treated and untreated	R/M HNSCC -	Demonstrated manageable toxicity, with promising anticancer activity for both previously treated and untreated patients.	[271]
Combination of Avelumab, Cetuximab, and Palbociclib	Phase 1 clinical trial 1st line	R/M HNSCC -	Showed good tolerability and clinical responses.	[272]
Combination of Cetuximab and Pembrolizumab	Phase 2 clinical trial ≥1st line	R/M HNSCC -	Improved ORR and slightly increased toxicity when compared to single-drug therapies.	[273]
Afatinib in combination with Pembrolizumab	Phase 2 clinical trial ≥1st line	R/M HNSCC -	The combination improved ORR.	[274]
Cetuximab combined with Nivolumab	Phase 1/2 clinical trial 2nd line	R/M HNSCC -	Exhibited good safety; however, no improvement of OS was observed.	[275]
Combination of Vorinostat and Pembrolizumab	Phase 2 clinical trial -	R/M HNSCC and SGC -	Exerted synergistic effects, increasing anticancer activity. Higher toxicity than Pembrolizumab monotherapy.	[276]
Resveratrol combined with PD-L1 Inhibition	Preclinical trial	N/A	Exhibited in vitro and in vivo antiproliferative effects.	[277]
Atezolizumab plus Cobimetinib	Phase 2 clinical trial -	HNSCC -	Moderate activity was observed for patients who had not previously been treated with PD-1/PD-L1 inhibitors.	[281]
Navoximod combined with Atezolizumab	Phase 1 clinical trial -	HNSCC -	Showed acceptable safety and tolerability profile and antitumoral activity, but there was no evidence of a benefit of the combination.	[284]
Epacadostat combined with Pembrolizumab	Phase 1/2 clinical trial -	HNSCC -	Exhibited good safety and moderate anticancer response.	[285]

Abbreviations: CTLA-4, cytotoxic T-lymphocyte-associated protein 4; HNSCC, head and neck squamous cell carcinoma; IC; induction chemotherapy; LRR HNSCC, locoregionally recurrent head and neck squamous cell carcinoma; N/A, not applicable; OCSCC, oral cavity squamous cell carcinoma; ORR, objective response rate; OS, overall survival; PD-1, programmed cell death protein 1; PFS, progression-free survival; R/M HNSCC, recurrent/metastatic head and neck squamous cell carcinoma; SGC, salivary gland cancer; TPF, docetaxel, cisplatin, and fluorouracil; -, data not provided.

### 3.6. Microtubule Inhibition-Based Combination Therapies

Microtubules are dynamic, hollow cylindrical structures composed of tubulin protein subunits, which play a critical role in cellular processes. In the context of anticancer therapy, microtubules are an important target due to their crucial involvement in cell division. During mitosis, microtubules undergo dynamic assembly and disassembly, forming the mitotic spindle which is essential for accurate chromosome segregation. Disruption of microtubule dynamics interferes with this process, leading to cell cycle arrest, mitotic catastrophe, and subsequent cell death [286,287].

Antimicrotuble drugs, such as Paclitaxel and Docetaxel, are commonly used in HNSCC treatment in combination with 5-FU and platinum-based drugs. These antimitotic drugs promote the activation of the spindle assembly checkpoint (SAC) by disrupting the polymerization dynamics of microtubules [22]. The activation of SAC leads to prolonged mitotic arrest that can cause apoptosis induction or lead to an arrest in a senescence-like G1 state [288]. Cancer cells have a higher rate of proliferation, making them the perfect target for suppression of microtubule dynamics since the mitotic stage is a particularly vulnerable state with faster microtubule dynamic activity [289]. Thus, combinatorial approaches focusing on antimitotic drugs have been widely explored in clinical trials (Table 7) [290].

In ovarian cancer, overexpression of activated AKT reduces Paclitaxel-induced apoptosis, which can be reverted through inhibition of PI3K [291]. A phase 2 trial with Buparlisib and Paclitaxel in platinum-pretreated R/M HNSCC patients revealed an increase in PFS, OS, and ORR when compared with Paclitaxel alone. Nonetheless, an increase in grade 3/4 adverse events was also reported. This combination could be used as a second-line treatment for platinum-pretreated R/M HNSCC patients, although further investigation is needed to confirm these findings [292]. However, a similar combination with Docetaxel and PX-866 in R/M HNSCC patients showed no improvement in PFS, ORR, or OS in comparison with Docetaxel alone [293].

Combinatorial approaches with Paclitaxel and drugs targeting DNA have also been explored. For instance, a phase 2 trial with biweekly Gemcitabine and Paclitaxel in HNSCC patients reported a median PFS of 4 months, an OS of 8 months, an ORR of 28%, and no treatment-related deaths [294]. This regimen was considered safe and had satisfactory efficacy, and can be an option for patients that cannot receive platinum-based chemotherapy [294]. In a phase 2 trial in LA-HNSCC genexol-PM, a preparation of Paclitaxel without Cremophor EL (CrEL), and Cisplatin were combined and administered as IC. CrEL induces histamine release and is possibly the cause of hypersensitivity reactions. Genexol-PM has demonstrated promising antitumor activity, with some reports of superior efficacy when compared to Paclitaxel, and good tolerability. Furthermore, it was approved in Korea for the treatment of various types of cancer [295,296]. The combination led to tumor shrinkage in 48 of the 52 patients in this study and a DCR of 94.2%. The endpoint ORR of 70% was not met, and the PFS and OS were not reached [296]. Addition of Temsirolimus to low-dose weekly Carboplatin and Paclitaxel in R/M HNSCC patients showed a manageable toxicity profile and an ORR, a PFS, and an OS of 41.7%, 5.9 months, and 12.9 months, respectively. The enhanced efficacy of this combination is possibly due to the synergistic activity between mTORC1 inhibition and cytotoxic chemotherapy [297]. The addition of Docetaxel to a Cetuximab, 5-FU, and Cisplatin regimen, intended to improve the efficacy of the treatment in R/M HNSCC patients, led to disappointing results since a high mortality rate and toxicity were detected [298].

The survival of p53-mutant HNSCC cells is dependent on several G2/M checkpoint regulator genes, including WEE1, making them interesting targets since this is not observed for healthy cells [299]. Combining a WEE1 inhibitor with Cisplatin, Gemcitabine or Carboplatin in patients with advanced solid tumors was deemed safe [300]. Additionally, the combination with weekly Docetaxel and Cisplatin was considered safe and tolerable, and encouraging antitumor efficacy in advanced HNSCC was noted. Moreover, a reduction in pY15-CDK and exacerbated apoptotic signaling were observed in patients that responded to treatment [299]. Phosphorylation of Y15 is promoted by WEE1 leading to the inhibition of CDK1 and, consequently, premature mitosis and cell death [301,302]. WEE1 inhibitors were also combined with PARP inhibitors for TSCC cell lines but rarely showed synergistic effects [224].

Combinatorial approaches with antimitotic drugs were mainly focused on improving therapeutic combinations already in use. Combinatorial approaches with antimitotic drugs were mainly focused on improving therapeutic combinations already in use, showing promising outcomes.

**Table 7:** Main combination therapies based on microtubules targeting used in clinical trials for oral cancer treatment.

Combination Therapies	Study Design/Treatment Type	Cancer Type and Stage	Main Reported Outcome	References
Combination of Buparlisib and Paclitaxel	Phase 2 clinical trial 2nd line	R/M HNSCC -	Increased PFS, OS, and ORR when compared to single paclitaxel therapy; however, higher toxicity was observed.	[292]
Combination of Docetaxel and PX-866	Phase 2 clinical trial 2nd and 3rd line	R/M HNSCC -	Exerted no improvement in PFS, ORR, or OS when compared to docetaxel monotherapy.	[293]
Gemcitabine combined with Paclitaxel	Phase 2 clinical trial 1st line	R/M HNSCC -	Exhibited satisfactory efficacy and good safety, with no treatment related deaths.	[294]
Combination of Cisplatin with Genexol-PM	Phase 2 clinical trial 1st line (IC)	LA-HNSCC III/IVa/IVb	Promoted tumor reduction in 48 of the 52 patients	[296]
Combination of Temsirolimus with low-dose weekly Carboplatin and Paclitaxel	Phase 2 clinical trial ≥1st line	R/M HNSCC -	Exerted synergistic effects, with manageable toxicity profile.	[297]
Combination of Docetaxel, Cetuximab, 5-FU, and Cisplatin	Phase 2 clinical trial 1st line	R/M HNSCC -	Did not improve efficacy and showed high toxicity profile and mortality rate.	[298]
AZD1775 combined with Cisplatin, Gemcitabine, or Carboplatin	Phase 1 clinical trial -	HNSCC -	Exhibited good tolerability in patients with advanced solid tumors.	[300]
AZD1775 combined with neoadjuvant weekly Docetaxel and Cisplatin	Phase 1 clinical trial -	LA-HNSCC III/IVb	Exhibited a good safety, efficiency, and tolerability profile.	[299]
Combination of WEE1 and PARP inhibitors	Preclinical trial	N/A	Exerted no synergistic effects	[224]

Abbreviations: HNSCC, head and neck squamous cell carcinoma; IC; induction chemotherapy; N/A, not applicable; ORR, objective response rate; OS, overall survival; PFS, progression-free survival; R/M HNSCC, recurrent/metastatic head and neck squamous cell carcinoma; -, data not provided.

### 3.7. Other Target Inhibition-Based Combination Therapies

Since minimal improvements have been made regarding HNSCC patients’ survival rates, there is a need for different approaches who can be useful for patients that do not respond to the available treatment options. In this sense, several distinct strategies have been evaluated for the treatment of oral cancer, with less often explored targets that will be described below (Table 8).

Induction of apoptosis is a common therapeutic strategy for cancer. Apoptosis can be promoted through two distinct pathways: the intrinsic/mitochondrial pathway and the extrinsic pathway induced by death receptor (DR) signaling [303]. TNF-related apoptosis-inducing ligand (TRAIL) is a TNF family member that binds to DR4 and/or DR5 expressed on the surface of cancer cells and induces apoptosis. In contrast, normal cells do not express these receptors, making it, in theory, a safer and more specific treatment [304]. However, TRAIL has a short biological half-life, and cancer cells can develop resistance to this treatment [305,306]. α-Mangostin, a xanthone that promotes cell-cycle arrest and apoptosis in cancer cells, was shown to sensitize colon cancer cells to TRAIL treatment by increasing DR5 expression and inducing cell surface display of these receptors [307]. In OSCC, it was found that TRAIL enhances α-Mangostin-mediated apoptosis and suppresses cell proliferation [304].

Ascorbic acid (AA) has also been shown to sensitize cells to chemotherapy [308,309]. Interaction with several metal ions leads to the auto-oxidation of AA. The resulting ascorbate radical is a crucial intermediate in hydrogen peroxide (H_2_O_2_) formation. H_2_O_2_ is the driver of AA cytotoxicity by compromising cell integrity and metabolism [310]. From different combinations of solid lipid nanoparticles tested in vivo, Paclitaxel in combination with AA was the most effective treatment, leading to moderate dysplasia and minimal display of in situ carcinoma, suggesting it might be a promising treatment for oral cancer [9].

Heteronemin is a marine terpenoid that exerts anticancer activity in several types of cancer with low cytotoxicity to healthy cells [311,312,313]. Its anticancer activity arises from the induction of oxidative stress by producing ROS while also inhibiting expression of Bcl-X_L_, Bcl-2, cyclin D1, and p53 [312,313]. Recently, it was reported that Heteronemin can reduce proliferation in oral cancer cells through inhibition of ERK1/2 and STAT3 [10]. It also inhibits Thrombospondin 1 (THBS-1) while suppressing TGF-β expression in OEC-M1 cells. The thyroid hormone deaminated analog, 3,3′,5,5′-tetraiodothyroacetic acid (Tetrac), showed antiproliferative activity in cancer cells, with low cytotoxicity to normal cells, by activation of tumor-suppressor genes, such as p53, while inhibiting proliferative genes [10]. In OSCCs the combination of Heteronemin and Tetrac exerts a synergistic anticancer effect by inhibiting ERK1/2 activation, and suppressing THBS-1 and TGF-β expression while promoting p53 phosphorylation.

Nevertheless, cancer cells usually suppress the mitochondrial apoptosis pathway through mutations or deletions of p53 [314]. The protein p53 binds to prosurvival members of the BCL-2 family, such as BCL-X_L_, BCL-w, and BCL-2, suppressing their apoptosis inhibitory function [315]. MCL-1 is also a prosurvival member of this family overexpressed in various tumors, and is associated with a poor prognosis and the development of resistance to treatment [316]. In HNSCC patients, MCL-1 is a predictor of radiotherapy and chemotherapy responsiveness [314]. MCL-1 activity is regulated by a pro-apoptotic protein called NOXA [317]. A combination of Navitoclax, a BCL-2 and BCL-X_L_ inhibitor, and a NOXA inducer, evaluated in OSCCs, promoted cell death independently of p53 activation. It was also suggested that MCL-1 and BCL-X_L_ inhibition was sufficient for apoptosis induction [314]. In addition, MCL-1 expression can potentially lead to resistance to Navitoclax therapy. Thus, a combination with Navitoclax and an MCL-1 inhibitor was also explored, showing synergistic effects. The study also showed that Navitoclax does not radiosensitize HNSCC cells [318].

As stated above, autophagy is involved in tumor development and therapy resistance. PI3K signaling plays a role in autophagy regulation. In several types of cancer, the combination of a PI3K inhibitor and autophagy inhibition resulted in antiproliferative activity and decreased tumor xenografts growth [319,320]. In HNSCC cell lines, an antiproliferative synergistic effect, independent of *PIK3CA* status, and blockage of autophagy induced by PI3K inhibition, were observed [321].

The combination of an IGF-1R inhibitor with an Src inhibitor showed synergistic activity in HNSCC cell lines through focal adhesion kinase (FAK) inhibition. FAK is involved in cell migration and invasion. Its inhibition alone leads to a reduction in HNSCC cell growth and can induce apoptosis [322].

Nanoparticles have been used to reduce cytotoxicity and deliver a constant drug concentration for a longer period. Nanoparticle albumin-bound Paclitaxel in combination with Cetuximab and Carboplatin for R/M HNSCC, in comparison with the EXTREME regimen, revealed improved ORR, OS, and depth of response (CR rate), defined as the maximal tumor shrinkage observed. However, PFS showed no improvement [278,323]. In this approach, albumin was used to target cancer cells with upregulated macropinocytosis, a pathway that is associated with EGFR, RAS, and PIK3 signaling pathways [279]. When compared to Pembrolizumab as monotherapy or in combination with chemotherapy, this approach also had a higher ORR, CR rate, and median OS; however, the duration of response was inferior. Treatment with Irinotecan, an inhibitor of DNA topoisomerase I, was shown to activate NF-κB in tumor-bearing mice, which can lead to resistance to Irinotecan treatment [324]. NF-κB is involved in pro-tumoral activity. Evidence from human non-small cell lung cancer cells showed that Irinotecan combined with a proteasome inhibitor improved Irinotecan anticancer activity and increased IκB-α, an NF-κB inhibitor protein, expression [324,325]. This combination revealed disappointing results in R/M HNSCC patients, showing a lower OS than other treatment options [326].

Notch is involved in cell proliferation and metabolism, partially through the PI3K pathway [327]. γ-secretase is a protease that is involved in the activation of Notch signaling [328]. Thus, a combinatorial approach inhibiting both Notch and PI3K signaling should lead to enhanced PI3K pathway inhibition. In a phase 1 trial with patients with advanced solid tumors, 3 out of 15 HNSCC patients responded to the combinatorial treatment. However, 42% of patients experienced grade 3 adverse events, and, thus, the study was terminated [327].

Motolimod is a TLR 8 agonist associated with enhancement of antitumor immunity and increase of ADCC [329]. However, combining Motolimod with the EXTREME regimen in HNSCC patients induced no improvement of PFS and OS, but was considered safe. Nevertheless, HPV-positive patients with oropharyngeal cancers, and participants with injection site reactions, had better responses and longer PFS and OS. HPV-positive patients possibly showed a better response due to enhanced immune responses within the tumor microenvironment [330].

Lysine-specific demethylase 1 (LSD1) is involved in the regulation of transcription and maintenance of pluripotency of stem cells [331]. Overexpression of LSD1 has been associated with resistance to treatment in several types of cancer while its inhibition leads to the promotion of antitumor immunity [331,332,333]. Thus, combinations of an LSD1 inhibitor with a YAP, involved in pro-tumorigenic gene expression, inhibitor or with PD-1 and PD-L1 inhibitors were explored. The YAP combination showed antiproliferation activity in vitro and addictive effects in vivo. LSD1 inhibition led to the upregulation of PD-L1 and the concurrent treatment in vivo showed decreased tumor growth and increased T-cell infiltration [334].

Hypoxia in tumor microenvironments is associated with resistance to treatment and it is regulated by HIFs such as HIF-1α [335]. The thioredoxin system is usually overexpressed in tumor cells since it acts as a defense system against ROS [336]. Thioredoxin-1 (Trx-1) is part of this system and was shown to have also antiapoptotic activity [337]. Vorinostat affects HIF-1α stability and increases ROS production while PX-12, a Trx-1 inhibitor, indirectly influences HIF-1α activity. Thus, concurrent administration of both inhibitors in OSCC showed a synergistic effect under hypoxia but addictive effects in normoxia. A possible explanation is the fact that HIF-1α is active during hypoxic conditions whereas it is degraded in normoxic ones [335].

These less explored approaches led, in general, to promising results in preclinical studies. However, clinical trials with less common combinatorial approaches showed mostly disappointing results. Nonetheless, alternative approaches should continue to be explored to expand therapeutic options for patients who do not respond to conventional ones.

**Table 8:** Other combination therapies targeting different cellular components used in clinical and preclinical trials for oral cancer treatment.

Combination Therapies	Study Design/Treatment Type	Cancer Type and Stage	Main Reported Outcome	References
Combination of α-Mangostin with TRAIL treatment	Preclinical trial	N/A	Inhibited cell proliferation and promoted tumor apoptosis in OSCC.	[304]
Solid lipid nanoparticles containing Paclitaxel and AA	Preclinical trial	N/A	Exerted high efficacy, leading to moderate dysplasia in vivo.	[9]
Combination of heteronemin with tetrac	Preclinical trial	N/A	Exerted synergistic effects potentiating anticancer activity.	[10]
Navitoclax combined with NOXA inducer	Preclinical trial	N/A	Efficiently promoted HNSCC cell death by apoptosis.	[314]
Navitoclax combined with MCL-1 inhibitor	Preclinical trial	N/A	The combination exhibited synergistic activity.	[318]
Combination of PI3K and autophagy inhibitors	Preclinical trial	N/A	Exerted synergistic effects, decreasing cancer cells proliferation.	[321]
Combination of an anti-IGF1R with an Src inhibitor	Preclinical trial	N/A	Exerted synergistic effects, enhancing anticancer response in HNSCC cells.	[322]
Nanoparticle albumin-bound Paclitaxel combined with Cetuximab and Carboplatin	Phase 2 clinical trial 1st line	R/M HNSCC -	Improved ORR and OS and induced tumor reduction; however, no improvement in PFS was observed.	[323]
Irinotecan combined with Bortezomib	Phase 2 clinical trial -	LA-HNSCC -	Reduced OS when compare with other therapies.	[326]
Combination of Ridaforolimus and MK-0752	Phase 1 clinical trial -	R/M HNSCC -	Showed activity in HNSCC patients, but considerably increased the side effects in patients with advanced solid tumors.	[327]
Combination of Motolimod with the EXTREME regimen	Phase 2 clinical trial 1st line	R/M HNSCC -	Exerted good safety; however, no improvements of PFS and OS in HNSCC patients were observed. Led to enhanced outcomes for HPV-positive oropharyngeal cancer patients.	[330]
Combination of LSD1 and YAP inhibition	Preclinical trail	N/A	Exerted additive effects in inhibiting cell proliferation.	[334]
Combination of HIF-1α and Trx-1 inhibitor	Preclinical trail	N/A	Exerted synergistic effect under hypoxia condition and addictive effects in normoxia.	[335]

Abbreviations: AA, Ascorbic acid; HNSCC, head and neck squamous cell carcinoma; N/A, not applicable; OS, overall survival; OSCC, oral squamous cell carcinomas; PD-L1, programmed cell death ligand 1; PFS, progression-free survival; R/M HNSCC, recurrent/metastatic head and neck squamous cell carcinoma -, data not provided.

## 4. Conclusions and Future Perspectives

In several of the preclinical and clinical trials discussed in this review, the importance and need for therapeutic approaches to consider the specific characteristics of the patient and the tumor are evident. This highlights the importance of analyzing the best options for each individual and the necessity for a wide range of treatment options.

The most common reasons for combinatorial approaches to fail in the clinical trials analyzed were high toxicity/safety concerns or/and lack of efficacy/clinical activity and benefits. It is suggested that this lack of efficacy/clinical activity and benefits may be due to possible redundant pathways that may overcome the action of some inhibitors. Another reason may be that in some trials, patients were not selected for the specific targets explored in the combinatorial approaches, masking a possible benefit for some subpopulations of HNSCC. In addition, trials with a small number of patients may not provide a comprehensive evaluation of clinical significance. The limited clinical efficacy of some combination therapies compared to monotherapy in oral cancer can be attributed to several underlying reasons. Firstly, the complex and heterogeneous nature of oral tumors is considered a challenge in effectively targeting multiple pathways simultaneously. Additionally, the emergence of drug resistance mechanisms within the tumor microenvironment contributes to the reduced effectiveness of combination therapies. Furthermore, the pharmacokinetics and pharmacodynamics of multiple drugs in combination can lead to unfavorable interactions, impacting their efficacy. Drug–drug interactions, variations in drug metabolism, and differing half-lives may affect the optimal dosing and exposure of each agent, potentially compromising their synergistic effects. To overcome these barriers, a multifaceted approach can be considered. Firstly, a comprehensive understanding of the molecular mechanisms underlying oral cancer and its resistance pathways is essential. This knowledge can guide the identification of predictive biomarkers and the development of patient stratification strategies, enabling tailored combination therapies based on individual tumor characteristics. In addition, incorporating immunotherapeutic strategies, such as immune checkpoint inhibitors or adoptive cell therapies, in combination regimens can harness the immune system to augment the antitumor response and overcome immune evasion mechanisms. Moreover, advancements in drug delivery technologies, such as nanoparticle-based systems or localized drug release platforms, can improve the spatiotemporal distribution and bioavailability of therapeutic agents within the tumor, thereby enhancing treatment efficacy. To address these challenges effectively, interdisciplinary collaborations among clinicians, researchers, and pharmaceutical companies are crucial. Integrating clinical trials with comprehensive molecular profiling, predictive biomarker discovery, and innovative drug delivery systems will facilitate the development of personalized combination therapies that can overcome resistance mechanisms and improve clinical outcomes in oral cancer patients.

An interesting observation of the currently active clinical trials in clinicaltrials.gov, which are exploring therapeutic combinations in HNSCC, is that the vast majority have as their main approach the combination of anti-PD1 or anti-PD-L1 drugs. We also observed that the most investigated therapeutic approaches in pre-clinical trials were those with drugs already approved for the treatment of HNSCC. These combinations were held mainly in order to try to overcome therapeutic resistances developed by tumor cells or improve the therapeutic outcome. Thus, in the near future, a similar trend should be observed with the recently approved drugs, Pembrolizumab and Nivolumab, or other drugs that target PD-1 or PD-L1.

However, there are multiple factors that need to be considered as prognostic markers for oral cancer, and these can play an important role in deciding the optimal combination therapy for each patient. Several clinical, histopathologic, and molecular characteristics that may be considered include (i) tumor stage and primary anatomic localization—this helps to determine how advanced the cancer is and to predict future metastasis; (ii) tumor thickness, pattern of invasion, histological grade, and margin status; (iii) tumor molecular-related biomarkers—expression of different molecules, some associated with tumor aggressiveness and patients’ poor survival, or give predictive response information on therapy use, and includes biomarkers, such as EGFR, p53, CD105, or HPV (mainly for oropharynx cancers); (iv) the patient’s overall health and medical history—factors such as age, existing medical conditions, nutrition, and lifestyle habits (smokers and alcohol drinkers) can all impact treatment options and outcomes [272,273]. The different existing therapeutic modalities for the treatment of oral cancer have advantages and disadvantages that must be weighed according to the individual characteristics of the patient.

Nonetheless, therapeutic combinations represent a promising way to fight oral cancer. Combining multiple therapies, including chemotherapy, radiation therapy, and immunotherapy, is expected to improve patient outcomes and reduce the risk of recurrence in the near future. The use of combination therapies has led to a reduction in the toxicity of individual treatments, allowing patients to tolerate more aggressive treatment regimens. The development of personalized medicine and targeted therapies will allow for even more precise and effective treatment of oral cancer. Finally, exploring the use of novel approaches, such as nanotechnology, gene therapy, and immunomodulation, is the right way to further improve treatment outcomes.

## Figures and Tables

**Table 4:** Main combination therapies based on CDK targeting used in clinical and preclinical trials for oral cancer treatment.

Combination Therapies	Study Design/Treatment Type	Cancer Type and Stage	Main Reported Outcome	References
Palbociclib combined with Afatinib	Preclinical trial	N/A	Induced cellular senescence and inhibited tumor growth.	[208]
Combination of Cetuximab with Ribociclib or Palbociclib	Preclinical trial	N/A	Exhibited less effectiveness than Ribociclib monotherapy in Cetuximab-resistant PDTX models. No improvement in anticancer response was observed when compared with Cetuximab alone.	[213]
	Phase 1 clinical trial -	R/M HNSCC -	Displayed good anticancer response, with tolerable side effects.	[214,215]
	Phase 2 clinical trial -	R/M HNSCC I–IV	Exhibited no significant improvement of OS and higher side effects than Cetuximab single therapy.	[216]
Palbociclib combined with Cetuximab	Phase 2 clinical trial Patients previously treated	R/M OPSCC -	Did not meet primary endpoint.	[217]
The combination of a CDK4/6 inhibitor with Suberanilohydroxamic acid	Preclinical trial	N/A	Enhanced inhibition of NPC cell growth in vitro and in vivo.	[218]
Palbociclib combined with Cisplatin	Preclinical trial	N/A	Exerted antagonistic effects, reducing cancer cell cytotoxicity.	[218]
Addition of Pablociclib and Carboplatin	Phase 2 clinical trial -	R/M HNSCC -	Insufficient antitumoral activity and high toxicity.	[220]
Combination of JQ1 with Palbociclib	Preclinical trial	N/A	Exerted synergistic effects, reducing tumor volume in vivo.	[221]
PI3K inhibitor combined with Palbociclib	Preclinical trial	N/A	Exhibited in vitro and in vivo controlled tumor growth.	[223]
Palbociclib combined with Dinaciclib	Preclinical trial	N/A	Exerted synergistic effects in tongue squamous cell carcinoma.	[207]
Dinaciclib combined with Cisplatin	Preclinical trial	N/A	Increased tumor growth inhibition in vivo.	[207]

Abbreviations: N/A, not applicable; NPC, nasopharyngeal carcinoma; OS, overall survival; PDTX, patient-derived tumor xenograft; R/M OPSCC, recurrent or metastatic oropharyngeal squamous cell carcinoma. -, data not provided.

**Table 5:** Main combination therapies based on BET protein targeting used in preclinical trials for oral cancer treatment.

Combination Therapies	Study Design	Main Reported Outcome	References
JQ1 combined with PI3K inhibitor	Preclinical trial	Exerted synergistic effects, increasing treatment efficacy in vivo and in vitro.	[228]
Combination of BRD4 and CDK7 inhibitors	Preclinical trial	Exhibited in vitro and in vivo synergistic effects.	[235]
Combination of a YAP inhibitor with melatonin	Preclinical trial	Exerted synergistic effects, increasing apoptosis and reducing metastatic activity.	[237]
JQ1 combined with siPD-L1	Preclinical trial	Improved in vitro and in vivo tumor growth inhibition.	[239]

Abbreviations: BRD4, bromodomain-containing protein 4; siPD-L1, small interfering RNA targeting PD-L1; YAP, Yes-associated protein.

## Data Availability

Not applicable.

## Abbreviations

5-FU: 5-Fluorouracil; AA: ascorbic acid; ADCC: antibody-dependent cellular cytotoxicity; Ang-1: angiopoietin-1; ATO: Arsenic trioxide; B7-H3: B7 homolog 3 protein; β-AR: β-adrenergic receptor; BCR: B-cell receptor; BET: bromodomain and extra-terminal domain; BRD4: bromodomain-containing protein 4; BTK: Bruton’s tyrosine kinase; CDKs: cyclin-dependent kinases; cIAP1/2: cellular inhibitor of apoptosis protein 1/2; cGMP: cyclic guanosine monophosphate; COX-2: cyclooxygenase-2; CrEL: cremophor EL; CTLA-4: cytotoxic T-lymphocyte-associated protein 4; DCR: disease control rate; DDR: DNA damage response; DNA-PK: DNA-dependent protein kinase; DR: death receptor; DSB: double-strand breaks; EGFR: epidermal growth factor receptor; FAK: focal adhesion kinase; FdUMP: fluorodeoxyuridine monophosphate; FdUTP: fluorodeoxyuridine triphosphate; FOXM1: forkhead box M1; FPR: favorable pathological response; FTI: farnesyltransferase inhibitor; FUTP: fluorouridine triphosphate; GMP: guanosine monophosphate; GTP: guanosine triphosphate; FGFR: fibroblast-growth-factor receptor; H_2_O_2_: hydrogen peroxide; HDAC: histone deacetylase; HER-2: human epidermal growth factor receptor type 2; HIF-1α: hypoxia-inducible factor 1 alpha; HLA-C: human leukocyte antigen-C; HNSCC: head and neck squamous cell carcinoma; HPV: human papillomavirus; IC: induction chemotherapy; IDO1: indoleamine 2,3-Dioxygenase 1; IFN-α: interferon alpha; IGF: insulin-like growth factor; IGF-1R: IGF type 1 receptor; IL-6: interleukin 6; KIR: killer immunoglobulin-like receptor; LA: locally advanced; LA-HNSCC: locally advanced head and neck squamous cell carcinoma; LA OPSCC: locally advanced oropharyngeal squamous cell carcinoma; LA OSCC: locally advanced oral squamous cell carcinoma; LRR: locoregionally recurrent head and neck squamous cell carcinoma; LSD1: lysine-specific demethylase 1; mAbs: monoclonal antibodies; MDSC: myeloid-derived suppressor cells; NFAT: nuclear factor of activated T cells; NF-κB: nuclear factor κB; NK: natural killer; NPC: nasopharyngeal carcinoma; OCC: oral cavity cancer; ORR: objective response rate; OS: overall survival; OSCC: oral squamous cell carcinomas; PARP: poly (ADP-ribose) polymerase; PD-1: programmed cell death protein 1; PDC: pyruvate dehydrogenase complex; PDE-5, phosphodiesterase-5; PDK, pyruvate dehydrogenase kinase; PD-L1: programmed cell death-ligand 1; PDTX: patient-derived tumor xenograft; PFS: progression-free survival; PKG: cyclic GMP-dependent protein kinase; PLA: polylactic acid; PTEN: phosphatase and tensin homolog; Rb: retinoblastoma protein; R/M HNSCC: recurrent/metastatic head and neck squamous cell carcinoma; R/M OPSCC: recurrent or metastatic oropharyngeal squamous cell carcinoma; RNAPII: RNA polymerase II; RTK: receptor tyrosine kinase; SAC: spindle assembly checkpoint; SASP: senescence-associated secretory phenotype; SDC: salivary duct carcinoma; SE: super-enhancer; SGC: salivary gland cancer; T4: thyroxine 4; Tetrac: 3,3′,5,5′-tetraiodothyroacetic acid, THBS-1: thrombospondin 1; TKIs: tyrosine kinase inhibitors; TLR: toll-like receptor; TNF: tumor necrosis factor; TPF: docetaxel, cisplatin, and fluorouracil; TRAIL: TNF-related apoptosis-inducing ligand; TRPV4: transient receptor potential vanilloid 4; Trx-1: thioredoxin-1; TS: thymidylate synthase; TSCC: tonsillar squamous cell carcinoma; VEGF: vascular endothelial growth factor; VEGFR: vascular endothelial growth factor receptor; XIAP: x-linked inhibitor of apoptosis protein; YAP: yes-associated protein.
